# Contemporary status of insecticide resistance in the major *Aedes* vectors of arboviruses infecting humans

**DOI:** 10.1371/journal.pntd.0005625

**Published:** 2017-07-20

**Authors:** Catherine L. Moyes, John Vontas, Ademir J. Martins, Lee Ching Ng, Sin Ying Koou, Isabelle Dusfour, Kamaraju Raghavendra, João Pinto, Vincent Corbel, Jean-Philippe David, David Weetman

**Affiliations:** 1 Oxford Big Data Institute, Li Ka Shing Centre for Health Information and Discovery, University of Oxford, Oxford, United Kingdom; 2 Institute of Molecular Biology and Biotechnology, Foundation for Research and Technology-Hellas, Heraklion, Greece; 3 Department of Crop Science, Pesticide Science Lab, Agricultural University of Athens, Athens, Greece; 4 Laboratório de Fisiologia e Controle de Artrópodes Vetores, Instituto Oswaldo Cruz, Fundação Oswaldo Cruz (FIOCRUZ), Manguinhos, Rio de Janeiro, Rio de Janeiro, Brazil; 5 Environmental Health Institute, National Environment Agency, Helios Block, Singapore; 6 Unité d'Entomologie Médicale, Institut Pasteur de la Guyane, Cayenne, French Guiana; 7 Insecticides and Insecticide Resistance Lab, National Institute of Malaria Research (ICMR), Delhi, India; 8 Global Health and Tropical Medicine (GHTM), Instituto de Higiene e Medicina Tropical (IHMT), Universidade Nova de Lisboa (UNL), Lisbon, Portugal; 9 Institut de Recherche pour le Développement (IRD), Maladies Infectieuses et Vecteurs, Ecologie, Génétique, Evolution et Contrôle (MIVEGEC), Montpellier, France; 10 Laboratoire d'Ecologie Alpine (LECA), Centre National de la Recherche Scientifique (CNRS), University Grenoble-Alpes (UGA), Grenoble, France; 11 Department of Vector Biology, Liverpool School of Tropical Medicine, Liverpool, United Kingdom; Johns Hopkins Bloomberg School of Public Health, UNITED STATES

## Abstract

Both *Aedes aegytpi* and *Ae*. *albopictus* are major vectors of 5 important arboviruses (namely chikungunya virus, dengue virus, Rift Valley fever virus, yellow fever virus, and Zika virus), making these mosquitoes an important factor in the worldwide burden of infectious disease. Vector control using insecticides coupled with larval source reduction is critical to control the transmission of these viruses to humans but is threatened by the emergence of insecticide resistance. Here, we review the available evidence for the geographical distribution of insecticide resistance in these 2 major vectors worldwide and map the data collated for the 4 main classes of neurotoxic insecticide (carbamates, organochlorines, organophosphates, and pyrethroids). Emerging resistance to all 4 of these insecticide classes has been detected in the Americas, Africa, and Asia. Target-site mutations and increased insecticide detoxification have both been linked to resistance in *Ae*. *aegypti* and *Ae*. *albopictus* but more work is required to further elucidate metabolic mechanisms and develop robust diagnostic assays. Geographical distributions are provided for the mechanisms that have been shown to be important to date. Estimating insecticide resistance in unsampled locations is hampered by a lack of standardisation in the diagnostic tools used and by a lack of data in a number of regions for both resistance phenotypes and genotypes. The need for increased sampling using standard methods is critical to tackle the issue of emerging insecticide resistance threatening human health. Specifically, diagnostic doses and well-characterised susceptible strains are needed for the full range of insecticides used to control *Ae*. *aegypti* and *Ae*. *albopictus* to standardise measurement of the resistant phenotype, and calibrated diagnostic assays are needed for the major mechanisms of resistance.

## Introduction

Arboviruses cause severe disease and death in humans with over 4 million disability-adjusted life years worldwide attributed to mosquito-borne viruses in 2013 [1, 2]. Furthermore, a marked increase in mortality and morbidity associated with dengue virus infections has been noted over the last decade in contrast to a drop in other major infectious diseases [3]. Each arbovirus is transmitted by multiple species and the relative importance of each species is determined by their vectorial capacity and their geographical distribution. Both *Aedes aegypti* and *Ae*. *albopictus* mosquitoes are competent vectors of 5 important arboviruses (chikungunya virus, dengue virus, Rift Valley fever virus, yellow fever virus, and Zika virus) and both are widely distributed, making these species an important factor in the worldwide burden of infectious disease. There are currently no registered vaccines for 3 of these arboviral diseases, and although the Dengvaxia vaccine for dengue has now been registered in several countries, it is not yet in widespread use. Yellow fever is the exception, with a long-standing vaccine that is used worldwide; however, funds for routine vaccination are limited [4]. In addition, prophylaxis options are not available for these diseases, meaning transmission is not interrupted during the human infection phase. Vector control by insecticides coupled with larval source reduction is therefore absolutely critical in the prevention and control of these arboviral diseases.

Control of *Aedes* vectors is currently primarily based on insecticides and community engagement for habitat management. The use of alternatives such as *Wolbachia* infection, sterile insect techniques, and genetic manipulation is promising but they have only been tested in restricted locations worldwide. The long lead time needed for new control measures means that current insecticide-based approaches will continue to play a central role for many years to come. Control of *Aedes* vectors using insecticides, primarily through larviciding or space spraying of pyrethroids and organophosphates, is fraught with complications including high cost, slow operational response, low community buy-in, and ineffective timing of application [5–7]. A further major concern is the rapid spread of insecticide resistance with the potential to reduce efficacy of current insecticide-based control strategies [8], but there is a lack of accessible data documenting the prevalence and mechanisms of insecticide resistance at specific geographical locations. These data are important to guide national programmes in the choice of the most effective compounds to use in each resistance setting. In this context, the Worldwide Insecticide Resistance Network (WIN), supported by the World Health Organization (WHO), was established to track insecticide resistance in the vectors of the arboviruses at a global scale and to evaluate the potential for deployment of alternative vector-control interventions [9]. Here, we review the current knowledge of insecticide resistance in *Ae*. *aegypti* and *Ae*. *albopictus* worldwide and assess underlying resistance mechanisms. Evidence on the global distribution of resistance to the major classes of neurotoxic insecticide is assessed and the tools available to monitor resistance phenotypes and genotypes are reviewed.

## Methods

### Database of bioassay records linked to geographical locations

A previously compiled database that searched the published literature up to October 2008 was provided by its authors [8]. We then searched the published literature from January 2009 to August 2016 using the Web of Knowledge database of published articles and the search terms “resistance” and “aedes”. Abstracts were scanned and full-text copies obtained for articles of potential relevance. Additional unpublished datasets were provided by members of the WIN. Data were extracted from all articles reporting either mortality or the lethal concentration required to kill half of the sample (LC_50_ values) from bioassays of *Ae*. *aegypti* and/or *Ae*. *albopictus* mosquito populations using 1 or more of the 4 major classes of neurotoxic insecticide (carbamates, organochlorines, organophosphates, and pyrethroids). Only data linked to a field collection at a defined site(s) with no more than 4 generations in the laboratory were included. Bioassays that used a synergist were also excluded. A PRISMA flow diagram of the full process and details of how these data were then mapped are provided in S1 File.

### Review of resistance mechanisms

Published studies of insecticide-resistance mechanisms were sought from Web of Knowledge using 3 sets of search terms: “*Aedes*” and (“pyrethroid” or “DDT”) and “resistance”; “*Aedes*” and (“organophosphate”, “temephos”, or “carbamate”) and “resistance”; and “*Aedes*” and (“kdr”, “P450”, “monooxygenase”, “glutathione”, or “esterase”). Because our focus was primarily to include studies capable of identifying specific genes and molecular markers associated with insecticide-resistance mechanisms, which can lead to diagnostic assays, studies solely investigating resistance mechanisms using biochemical assays or synergist tests were not reviewed unless complemented by more specific approaches. In studies of metabolic resistance, the number of genes identified as overexpressed in resistant populations or strains is highly variable, which in part relates to differences in the filtering strategies used to identify significance, and we have focused wherever possible on the genes or gene families for which replication was evident.

## Results and discussion

### The geographical distribution of the insecticide-resistance phenotype

A database of 6,888 bioassay records was compiled including 1,190 records from the 2008 review [8] and 5,698 new records. All data derived from published articles and those unpublished data for which we received permission to share are provided in S2 File, and the total number of data records for each class of insecticide resistance is given in Table 1.

**Table 1:** Number of bioassay records for each test type.

	Adult bioassays	Larval bioassays
***Aedes aegypti***		
**carbamates (*n* = 7)**		
susceptibility	163 (1978–2013)	0
dose response	5 (2009–2013)	80 (1965–2013)
**organochlorines (*n* = 4)**		
susceptibility	226 (1965–2014)	1 (2014)
dose response	29 (1967–2013)	189 (1958–2014)
**organophosphates (*n* = 12)**		
susceptibility	835 (1987–2016)	976 (1996–2014)
dose response	49 (2009–2016)	1,478 (1965–2015)
**pyrethroids (*n* = 17)**		
susceptibility	1,711 (1990–2016)	33 (2000–2011)
dose response	136 (2008–2016)	208 (1967–2014)
***Ae*. *albopictus***		
**carbamates (*n* = 4)**		
susceptibility	31 (1990–2013)	0
dose response	4 (1988–2013)	40 (2002–2011)
**organochlorines (*n* = 4)**		
susceptibility	49 (2003–2014)	4 (2014–2014)
dose response	2 (2013–2013)	37 (1965–1991)
**organophosphates (*n* = 11)**		
susceptibility	77 (1988–2016)	31 (1988–2014)
dose response	15 (1988–2016)	172 (1980–2013)
**pyrethroids (*n* = 12)**		
susceptibility	176 (1990–2016)	8 (2014–2014)
dose response	16 (1988–2016)	104 (1990–2011)

A record is defined as the mean mortality or the lethal concentration required to kill half of the sample (LC_50_ value) for a sample collected at a unique time and place by a unique study challenged with a unique insecticide. The year range spanned by each set of records is given in parentheses after the number of records. The number of different insecticides tested is given in parentheses after the insecticide class name.

**Fig 1:**
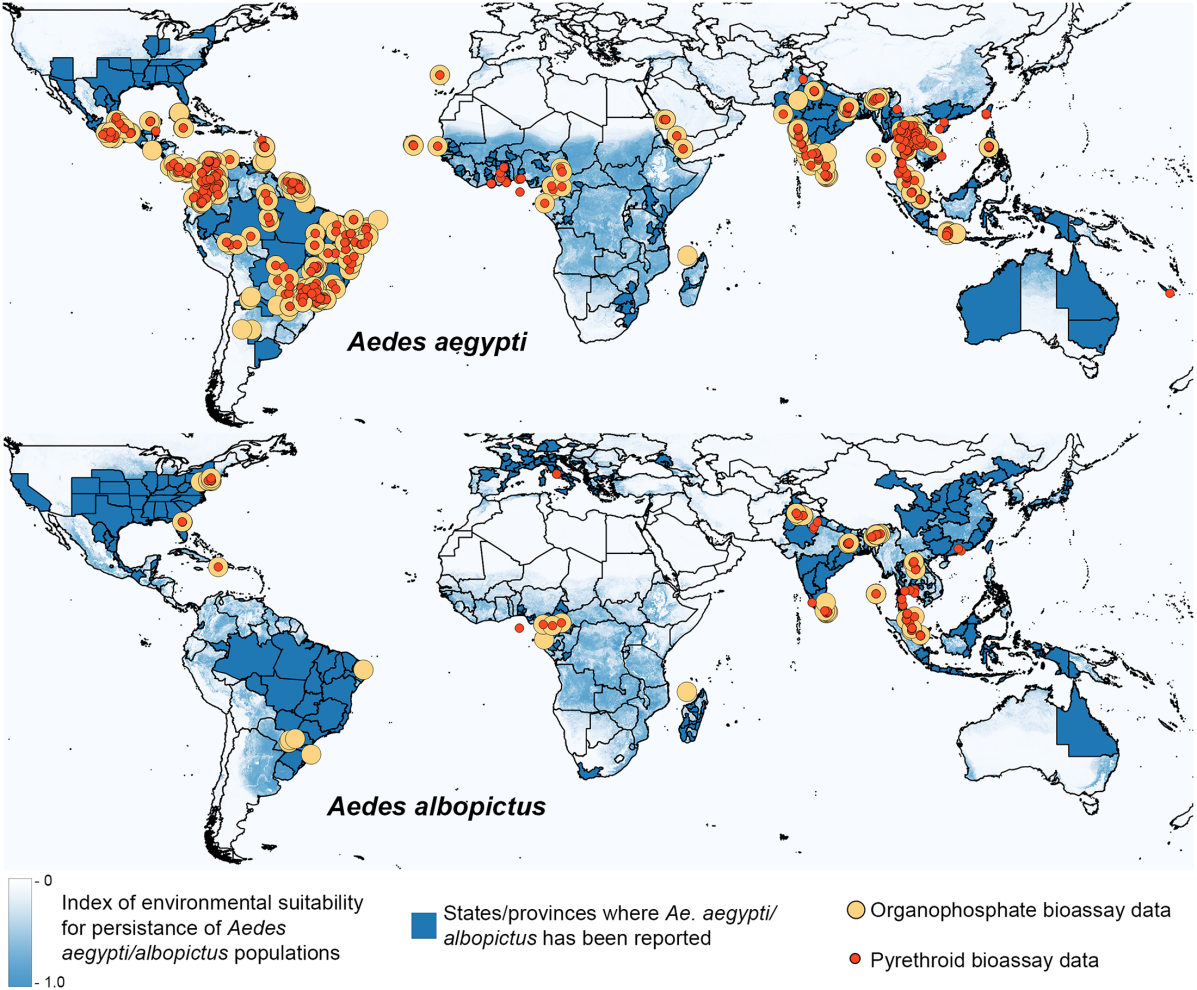
Locations of bioassay data for the organophosphates and pyrethroids, 2006 to 2015. Locations of populations that have been bioassayed (susceptibility and dose response, adult and larval) are shown for both insecticide classes, overlaid on maps of environmental suitability for *Ae*. *aegypti* and *Ae*. *albopictus* from Kraemer et al. (2015) eLife, 4: e08347.

**Fig 2:**
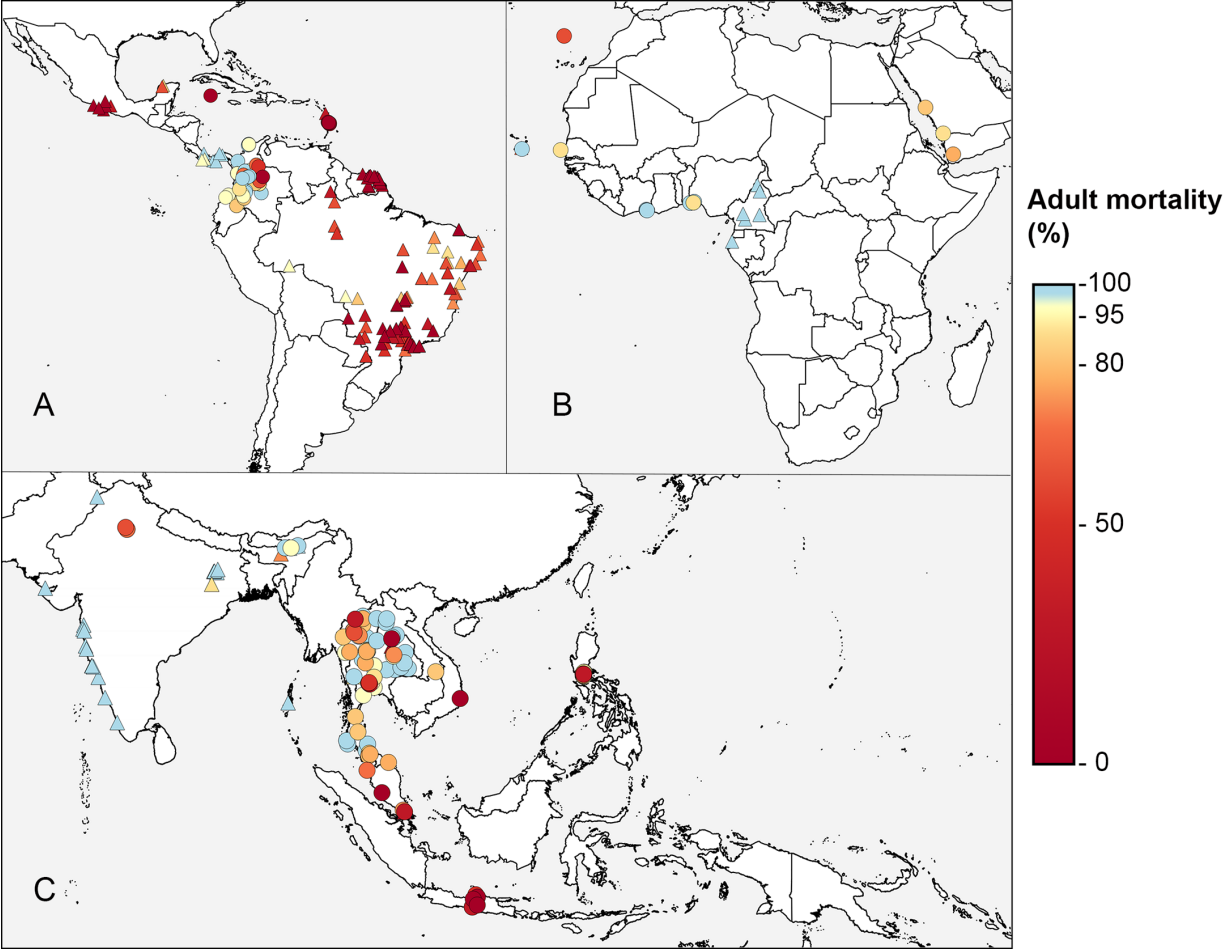
The frequency of resistance to deltamethrin in *Ae*. *Aegypti*, 2006–2015. Adult bioassays using 0.05% insecticide for 1 hour are denoted as circles and results from nonstandard adult bioassays (including different diagnostic doses and exposure periods) are denoted as triangles. The map is zoomed to the 3 regions with data. **(A)** Americas. **(B)** Africa/Arabian Peninsula. **(C)** Asia.

The geographical distributions of records of resistance/susceptibility to pyrethroids and organophosphates in *Ae*. *aegypti* and *Ae*. *albopictus* are shown in Fig 1. Good data coverage is evident for *Ae*. *aegypti* populations in much of the Americas and Asia but is lacking for Australasia and large parts of Africa. Less data is available for resistance to the main neurotoxic insecticides in *Ae*. *albopictus* with the exception of Southeast Asia. The amount of data available from the last decade is much greater than the amount available from the preceding decades, as shown in Fig B in S3 File. The data points shown on these maps, however, represent both susceptibility and dose-response bioassays performed on adults and larvae. To provide a more consistent measure of resistance, data for the most commonly used insecticide and test type were plotted and Fig 2 provides the results from bioassays that challenged adult *Ae*. *aegypti* with the most commonly tested pyrethroid, deltamethrin. These maps indicate that prevalence of resistance to deltamethrin is consistently high in Brazil and French Guiana and lower in the few locations in West Africa that have been sampled. Insecticide resistance appears to be highly variable or patchy in Southeast Asia. This may indicate that insecticide resistance is spatially heterogenous at a fine resolution but could also be the result of variation in test protocols, variation in collection dates, or small sample sizes. Apparent trends need to be treated with caution because these data do not come from a single study with a systematic sampling design and consistent methods. The dataset is highly biased, clustered in both time and space, and contains values generated using different methods.

**Fig 3:**
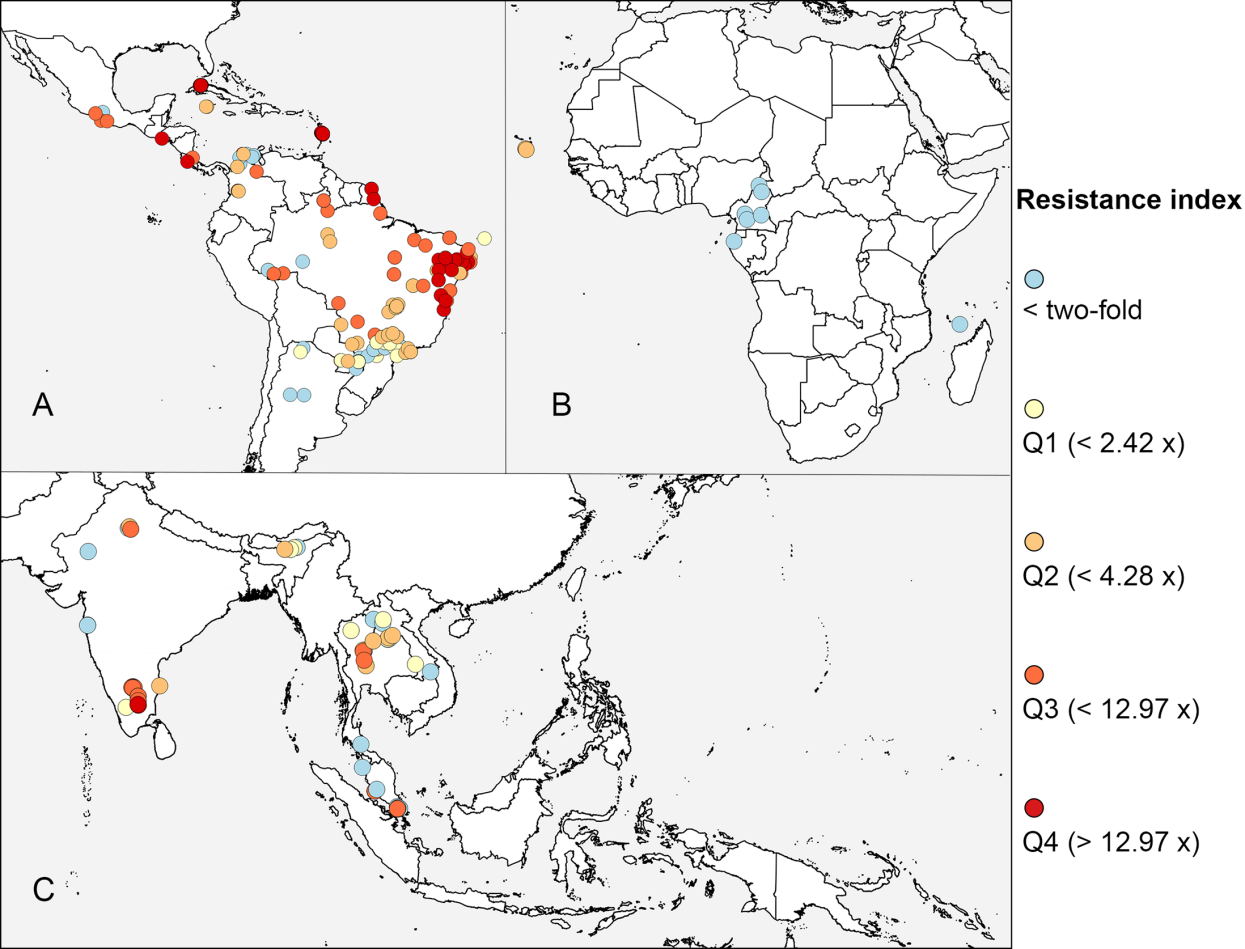
The level of *Ae*. *aegypti* resistance to temephos, 2006–2015. The ratio of the lethal concentration required to kill half of the sample (LC_50_ value) obtained by each study to the value obtained for the Rockefeller susceptible strain across studies was calculated. The ratios were then split into 5 classes: values less than 2-fold higher than Rockefeller and each quartile of the remaining distribution. The map is zoomed to the 3 regions with data. **(A)** Americas. **(B)** Africa. **(C)** Asia.

For *Ae*. *aegypti*, the most commonly tested organophosphate was temephos using larval diagnostic dose bioassays, and Fig 3 shows the distribution of results from these assays in each region for which data were available. No assumptions have been made about the levels of resistance that equate to “susceptible”, “possible resistance”, “moderate resistance”, “resistant”, or “highly resistant”, so the ratios obtained using the Rockefeller strain for comparison were classified into quartiles. The levels of resistance to temephos are higher in Brazil, French Guiana, and the Caribbean and lower at the few locations in West Africa that have been sampled. As seen for the pyrethroids, temephos resistance appears to be highly variable in southern and Southeast Asia. These apparent trends need to be assessed in the context of a number of potential confounding factors, as noted above.

**Fig 4:**
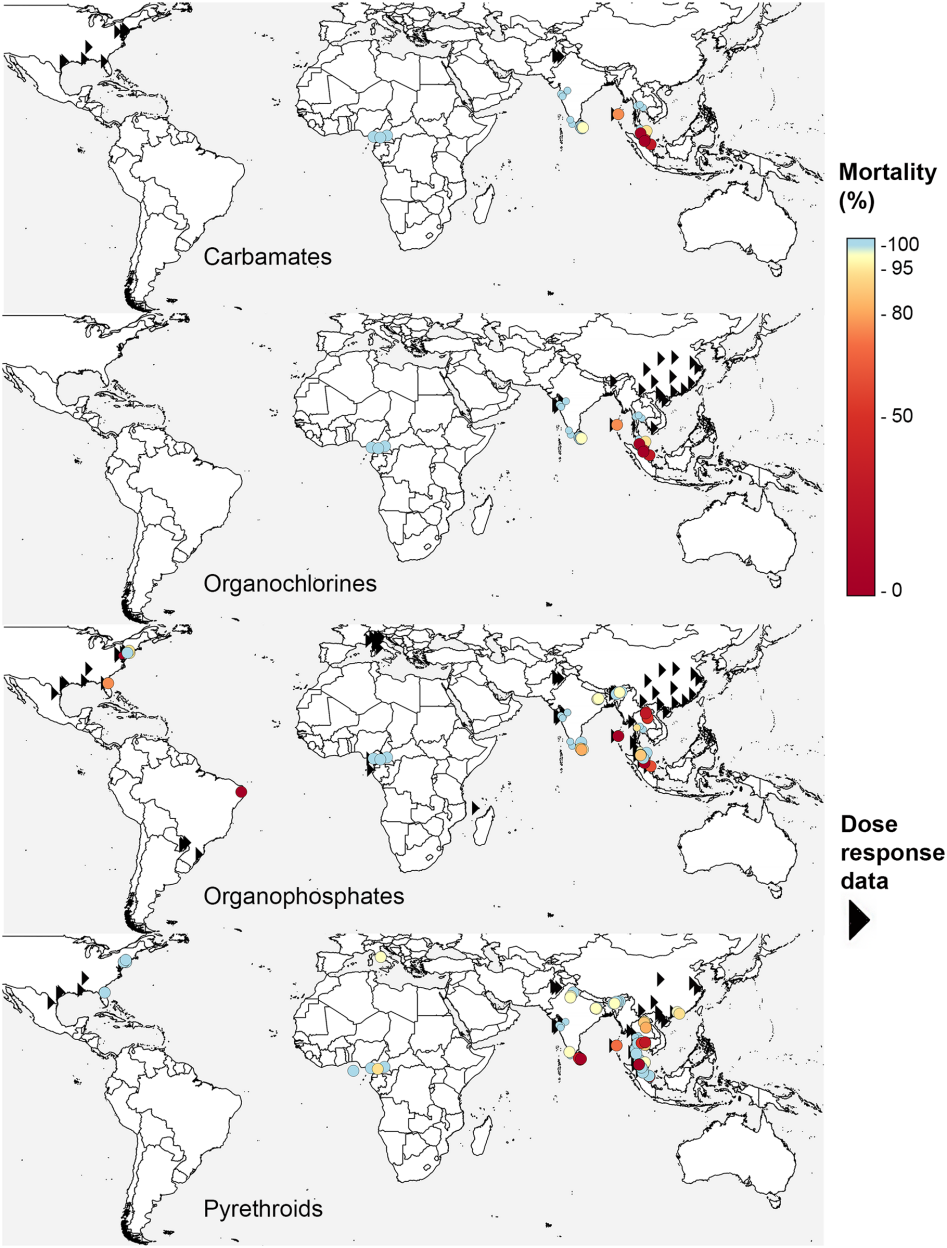
Frequency of insecticide resistance in *Aedes albopictus* in all years. The locations of *Ae*. *albopictus* populations used in susceptibility (circles) and dose-response (triangles) bioassays for each of the 4 main classes of neurotoxic insecticide. Both adult and larval bioassays are included. Mortality values for 2006–2015 are denoted by larger circles and the years up to 2005 are denoted by smaller circles.

For the other insecticide classes reviewed, resistance to the organochlorines is consistently high in *Ae*. *aegypti* populations whereas resistance to the carbamates is more variable, although evidence for resistance to the carbamates has been reported in Asia, Africa, and Latin America (Fig B in S3 File). Evidence for resistance to all 4 major classes of neurotoxic insecticides has been reported in *Ae*. *albopictus* populations from Southeast Asia, and resistance to the organophosphates has also been recorded in the Americas (Fig 4).

### Gaps in the insecticide-resistance phenotype data

A comparison of the respective distributions of *Ae*. *aegypti* and *Ae*. *albopictus* with the full insecticide-resistance datasets available for each species over the last decade (2006–2015) highlights those locations lacking contemporary data (Fig 1). For *Ae*. *aegypti*, there are very large gaps in the data available in Africa and far southeastern Asia/Australasia, in contrast to insecticide resistance in malaria vector species where data are available across much of Africa [10, 11]. Fewer data are available for *Ae*. *albopictus* and there are substantial data gaps on all continents. The maps in Fig 1 show the geographical spread of the data but combine multiple years and therefore mask any gaps in the temporal data coverage. The only region with good annual data coverage in the dataset presented here is Brazil (S2 File), although similar insecticide-resistance monitoring datasets may be held by other nations. In order to understand spatial heterogeneity and temporal trends, future analyses require good data coverage in both space and time and, ideally, standard measures of insecticide resistance. As shown in Table 1, there is currently considerable variation in the methods used to measure insecticide resistance in these 2 species and, in addition, Table A in S1 File shows that a diversity of strains with varying susceptibility have been used to calculate resistance ratios.

### Mechanisms of insecticide resistance

Before considering the geographical distribution of the most influential mechanisms of resistance, specific mechanisms need to be identified and validated. Mechanisms of resistance to insecticides in mosquitoes can include reduced penetration of insecticides into the insect, nonsynonymous mutations affecting the proteins targeted by insecticides (target-site mutations), or enhanced enzymatic biodegradation or sequestration (metabolic resistance) [12, 13]. Target-site mutations and metabolic resistance are thought to be the 2 main resistance mechanisms in *Aedes* mosquitoes. Reduced insecticide penetration is mainly caused by modifications of the insect cuticle, and though such changes have been identified in *Anopheles* species [14, 15], this remains poorly understood as a resistance mechanism in mosquitoes, and its importance in *Aedes* species is yet to be confirmed.

### Target-site resistance

The proteins targeted by chemical insecticides play a key role in the functioning of mosquito nervous systems. Any nonsynonymous mutation in these proteins can potentially decrease the affinity of insecticides to their target protein and contribute to resistance. Target-site mutations affecting all chemical insecticide classes are widespread in insects and are often well-conserved across taxa, permitting meaningful cross-species searches for mutations [16, 17].

#### Organophosphates and carbamates (acetylcholinesterase mutations)

Resistance to acetylcholinesterase (AChE) agonists is common in *Aedes* (Figs 3 and 4 and Fig B in S3 File) but AChE mutations do not occur widely [18–20]. Indeed, in contrast to *Anopheles* spp. and *Culex pipiens*, more than 1 mutation event is required to change glycine to serine at codon 119, the only commonly detected resistance-associated position in mosquitoes [17]. However, given the importance of the G119S mutation for resistance to carbamates and organophosphates [21, 22] and reports of organophosphate-resistant phenotypes in *Ae*. *aegypti* [23, 24] and *Ae*. *albopictus* [25, 26] coupled with recent detection of the 119S mutation in a population from India [27], continued monitoring of AChE mutations is important in *Aedes* populations.

#### Pyrethroids and DDT (voltage-gated sodium channel mutations)

**Fig 5:**
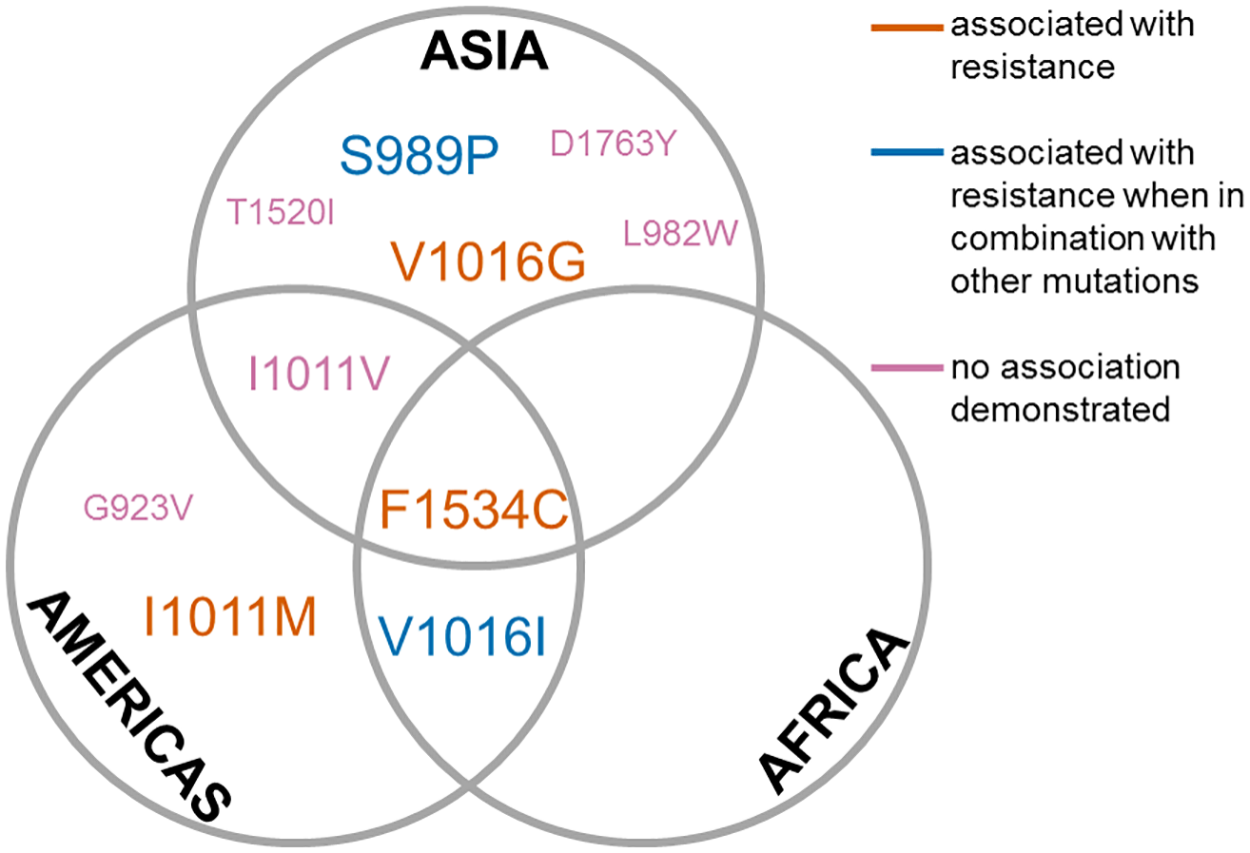
The geographical distribution of the 10 known voltage-gated sodium channel (VGSC) mutations in *Aedes aegypti* across the 3 continents in which they have been detected. Association of each mutation with pyrethroid resistance is shown in the key. Font size gives an indication of relative frequency.

Mutations in the voltage-gated sodium channel (VGSC), sometimes referred to as knockdown resistance (*kdr*) mutations, are very common in *Ae*. *aegypti*, with 10 mutations at 8 codon positions in VGSC domains II–IV, which have been found to comprise 15 haplotypes to date (Table A in S4 File). These mutations vary in frequency, geographical spread, and effects on resistance (Fig 5). The most widespread mutation in *Ae*. *aegypti*, 1534C, confers resistance to permethrin and deltamethrin when in combination with other mutations and is also linked with DDT resistance [28, 29]. It is found in both *Ae*. *aegypti aegypti* and *Ae*. *aegypti formosus* and occurs across 3 continents, with sequence data from the nearby short intron suggesting a single origin followed by worldwide spread [30]. In contrast, the V1016I and V1016G mutations have distinct geographical distributions, with 1016G found in Asia (spanning as far as Saudi Arabia) and V1016I in the Americas (although with recent detection at low frequency in Ghana) [30] (Table A in S4 File). Phenotypic effects of the 2 1016 mutations also differ. Prior to the discovery of F1534C, 1016I was thought to be a major cause of pyrethroid resistance [31, 32] but recent analyses, which also screened the F1534C position, found that the 1016I/1534C haplotype more strongly associates with pyrethroid resistance [33] and is increasing in frequency and range, whereas the 1016I/1534F haplotype is very rare [34, 35]. Thus, 1016I appears to enhance the pyrethroid-resistance phenotype conferred by 1534C rather than being directly causal, and at present in the Americas, only the F1534C and I1011M mutations appear capable of directly causing resistance [36]. The Asian variant V1016G can cause resistance alone but is more potent when in combination with another mutation, 989P, with which it commonly co-occurs (Table A in S4 File), and especially in a triple mutant 989P/1016G/1534C haplotype, which can engender extreme resistance [37]. Fortunately, 989P/1016G and 1534C usually occur on alternate chromosomes, but the triple mutant haplotype has been detected at low frequency in at least 3 distinct Asian locations (Table A in S4 File), likely created by independent recombination events. Careful monitoring of the occurrence and frequency of these 3 mutations in Asia is a high priority.

Although most commonly associated with positions 989, 1016, and 1534, other, less frequent mutations also occur in combinations: D1763Y with V1016G, G923V with I1011M, and T1520I with F1534C, and to date, their effects on resistance are unclear. Individually, VGSC mutations typically appear to be recessive in *Aedes* [38–40] but this can depend on their combination; for example, a S989P/V1016G/F1534C heterozygote genotype confers moderate resistance to deltamethrin and may present an important step in the evolution of high-level resistance or a genotype providing benefit across different pyrethroid classes [41].

In *Ae*. *albopictus*, only 4 VGSC mutations have been detected to date, affecting 2 codons at positions 1532 and 1534 (Table A in S4 File). The I1532T variant has only been detected in Italy to date but the 3 mutations at codon 1534 (F to C, L, or S) have broad distributions spanning multiple continents, which might reflect the rapid invasive spread of the species rather than multiple mutant origins. Only the F1534S variant has been demonstrated to be moderately associated with resistance to DDT and pyrethroids [42, 43]. Nucleotides at codons 989 and 1016 in the *Ae*. *albopictus* Folshan reference strain (VectorBase) are identical to the *Ae*. *aegypti* wild types, suggesting no intrinsic barrier to the evolution of elevated target-site resistance. Nevertheless, current evidence suggests that VGSC mutation is less common and, where present, may also link less strongly with pyrethroid resistance in *Ae*. *albopictus*.

### Metabolic resistance

Metabolic resistance is caused by elevated activity via overexpression or conformational change of enzymes that are involved in the processes of insecticide metabolism, sequestration, and excretion. Detoxification pathways may be complex and whilst the primary metabolic enzymes involved belong to large gene families (cytochrome P450 monooxygenases [P450s], glutathione S-transferases [GSTs], and carboxy/cholinesterases [CCEs]), other families are likely to be involved [13, 44]. Metabolic resistance is very common in mosquitoes and has been reported against all insecticides used in public health as well as agricultural pesticides that might be used for vector control in the future [44, 45].

#### Cyclodienes (gamma aminobutyric acid receptor mutation)

Despite withdrawal of the organochlorine insecticides targeting the gamma aminobutyric acid (GABA) receptor decades ago, the resistance to dieldrin (*Rdl*) mutation, A302S, has been detected in a cyclodiene-resistant strain of *Ae*. *aegypti* [46] and in wild populations of *Ae*. *albopictus* from La Reunion and Java [47, 48]. The persistence of the A302S mutation at high frequency might be selected by long-term environmental persistence of organochlorine pesticides or negligible fitness costs [49]. However, intriguingly, a recent electrophysiological study raised the possibility that there might be some contemporary insecticidal selection, with evidence for antagonism of the GABA receptor by both imidacloprid (a neonicotinoid) and deltamethrin, for which it may be a secondary target [50].

#### Pyrethroids

**Fig 6:**
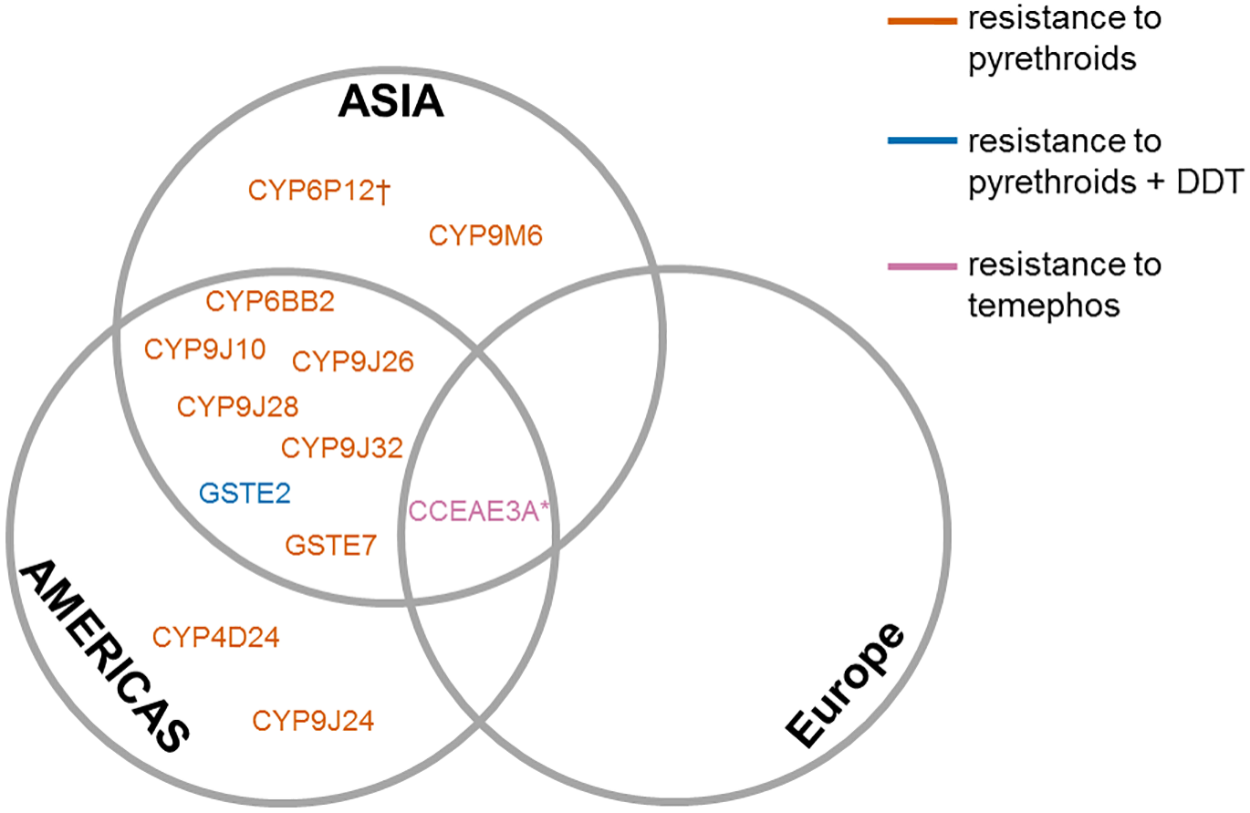
The geographical distribution of metabolic resistance detoxification genes based on significant overexpression. Genes are shown if linked to a resistance phenotype in transcriptomic studies, and a role has been demonstrated by functional validation (in vitro metabolism, ribonucleic acid interference (RNAi), or heterologous expression). All are in *Ae*. *aegypti* other than those marked: †*Ae*. *albopictus*; *both species.

Cytochrome P450s are involved in metabolism and detoxification of a wide range of compounds [51] and their overexpression is frequently associated with pyrethroid resistance in mosquitoes, although other detoxification enzymes are also implicated [12]. Multiple P450 genes, especially members of the CYP6 and CYP9 subfamilies, have been linked to resistance via overexpression in transcriptomic studies of insecticide-resistant versus susceptible strains (Fig A in S4 File). Whilst this suggests that a diversity of P450 genes may be involved, strains could be resistant to multiple insecticides or differ in other ways from susceptible colonies. Among the most consistently expressed genes, 4 (*CYP9J10*, *CYP6BB2*, *CYP9J26*, *CYP6J28*) have been proven to metabolise pyrethroids [52, 53] or to confer pyrethroid resistance when expressed transgenically in *Drosophila* [54]. In transcriptomic studies linking more closely with pyrethroid-resistant phenotypes, whilst there is substantial variation in results among strains within and among studies, both *CYP6BB2* and *CYP9J28* are detected as significant across studies (Table 2). The geographical distribution of results for functionally-validated genes is broader than is generally the case for VGSC mutations, with key candidate genes found to be significantly overexpressed across continents (Fig 6). In the sole study of *Ae*. *albopictus*, P450 genes again appear important, and although none of those overexpressed have been tested for metabolic activity, *CYP6P12* conferred pyrethroid resistance in transgenic *Drosophila melanogaster* [55].

**Table 2:** The main genes identified by transcriptomic studies specifically targeting expression responses to insecticide selection or exposure.

Species	Source	Selection	RR	Genes detected as overexpressed	Ref.
		**pyrethroids**			
*Aedes aegypti*	Cuba, Cayman	historical	30–1,000^A^	CYP9J9*, CYP9J10*, CYP9J26*, CYP9J27*, CYP9J28*, CYP6BB2*, 3 other P450 or related genes*, GSTE4*, ABCB4*, 2 UGT genes*	[43]
*Ae*. *aegypti*	Singapore	10 generations	1,650^B^	CYP9M4, CYP9M5, CYP9M6, CYP6Z7, CYP6Z8, **CYP6BB2**, CYP6F2, CYP6F3, 2 other P450 or related genes	[52]
*Ae*. *aegypti*	Mexico, Peru	5 generations	2.1–10.2^A^	CYP9J28*, CYP9J32*, CYP9J9*, 8 other P450 genes*, 2 CCE genes*, 2 GST genes*, aldo-keto reductase 4118*	[45]
*Ae*. *aegypti*	Bora Bora	10 generations	3.78^C^	aldo-keto reductase 4088	[46]
*Ae*. *albopictus*	Malaysia	exposure		CYP6P12, 16 other P450 genes, 2CCE genes, 5 GST genes, 1 UGT gene, 1 aldehyde oxidase gene, 11 cuticular protein genes, multiple other gene families	[47]
		**temephos**			
*Ae*. *aegypti*	Brazil	20 generations	175^D^	**CYP6N12**, **CCEAE3A**, GSTX2, **Aldehyde oxidase AO10382**	[48]
*Ae*. *aegypti*	Thailand	exposure	5.9–9.85^E^	**CCEAE3Α**, CCEAE6Α, 1 other CCE gene, **CYP6Z8**, **CYP9M9**, CYP6AH1, **CYP4H28**	[49]
*Ae*. *aegypti*	Colombia	exposure	15 ^A,E^	**CYP6N12**, CYP6M11, **CYP6F3**, 1 UGT gene	[15]
*Ae*. *aegypti*	Mexico, Peru	5 generations	42–390^A^	**CYP4H28, CYP6F3, CYP6Z8, CYP9M9**, 9 other P450 genes (8 CYP6 or CYP9), 8 GST genes, 10 CCE genes, **AO10382**, 7 other Redox genes	[50]
*Ae*. *albopictus*	Greece	12 generations	6.4^F^	CCEAE6A, **CCEAE3A**, 1 other CCE gene, CYP6M11, 7 other P450 genes, GSTX2, 1 other GST gene, 1 ABC gene, 5 UGT genes	[14]

Bold text denotes genes detected across studies; underlined text denotes genes detected across species.

The susceptible strain used to calculate the RR was New Orleans (A), SMK (B), Bora Bora (C), Rockefeller (D), Phatthalung (E), or a parental unselected line (F).

* genes significant in 2 or more comparisons.

**Abbreviations:** CCE, carboxy/cholinesterases; GST, glutathione S-transferases; P450, cytochrome P450 monooxygenases; RR, resistance ratio; Ref., Reference; UGT, UDP-glycosyltransferases

Whilst the majority of work has focused on gene expression, targeted sequencing of 760 candidate genes identified 55 nonsynonymous variants potentially linked to deltamethrin resistance in 10 P450 genes [56]. Studies of the association of expression of specific alleles of these genes with pyrethroid resistance in field populations are an important next step because the broad distributions of overexpression may have different underlying genetic causes, which also warrants studies testing allele specificity of metabolism. Interestingly, CYP6 and CYP9 genes were also the most commonly duplicated P450s, suggesting that copy number variation (CNV) plays an important role in differential expression phenotypes, and thus that design of informative DNA-based diagnostic assays could be possible.

#### Organophosphates

Quantitative trait locus mapping suggests the molecular underpinning of temephos resistance in *Ae*. *aegypti* is extremely multivariate [57]. This is supported by the diversity of genes highlighted in transcriptomic studies linking to temephos resistance, with multiple P450s, CCEs, and GSTs identified as overexpressed, although there is apparently greater consistency in the specific genes involved than for pyrethroid resistance (Table 2). This consistency also extends across species, with some common genes overexpressed in temephos-resistant *Ae*. *aegypti* and *Ae*. *albopictus*. Whilst the mode of action is unclear for most of these genes, CCEs catalyse the hydrolysis of ester bonds and have been frequently linked to both organophosphate and carbamate resistance, either through insecticide sequestration or direct metabolism [58]. In both *Ae*. *aegypti* and *Ae*. *albopictus*, the alpha-esterases *CCEAE3A* and *CCEAE6A* can be considered key candidates for temephos resistance, with up-regulation at least partially due to gene amplification. This is further supported by genetic crosses in *Ae*. *albopictus* that demonstrated a strong association between temephos survival and gene copy number [18, 59]. In both species, *CCEAE3A* can strongly sequester and slowly metabolize the temephos oxon and is a widespread resistance mechanism (Fig 6) present in American and Asian populations of *Ae*. *aegypti* and in *Ae*. *albopictus* populations from Europe and the United States of America [60].

#### Alternative gene families contributing to resistance

Although the mode of action of P450s in pyrethroid metabolism is better understood, other enzyme families may be involved. For example, CCEs can hydrolyse pyrethroids, with subsequent metabolism of the secondary metabolites by P450s [61]. Other detoxification genes are also implicated by ribonucleic acid interference (RNAi) knockdown in *Ae*. *aegypti*, notably *GSTE2* and *GSTE7* in deltamethrin resistance [62]. Epsilon GSTs are more commonly associated with resistance to DDT through direct dechlorination and their mode of action in pyrethroid-resistant *Ae*. *aegypti* resistance is currently unclear but might involve a peroxidase-activity protection from oxidative stress [63]. Gene families beyond the 3 major superfamilies are also likely to be important. For example, UDP-glycosyltransferases (UGTs), which can conjugate a glycosyl group to electrophilic substrates, are repeatedly detected as overexpressed in *Aedes* strains resistant to both pyrethroids and temephos (Table 2) and have been found amplified and affected by point mutations [56]. The activity of UGTs against hydrolysed pyrethroids has been demonstrated in rats [64] and activity-based probe fishing identified multiple UGTs interacting with pyrethroids [65]. Other enzymes potentially involved in insecticide resistance include adenosine triphosphate (ATP)-binding cassette (ABC) transporters, sulfotransferases, aldehyde oxidases, alcohol dehydrogenases, and short-chain dehydrogenases (Table 2). Further work is required to elucidate the role of each in the detoxification pathway.

### Resistance to alternative insecticides currently being deployed

*Bacillus thuringiensis* var. *israeliensis* (Bti) formulations and insect growth regulators (IGRs) are important alternatives to the neurotoxic insecticides for larviciding, in light of emerging widespread resistance to the latter. It has been proposed that Bti is resilient to the emergence of resistance due to multiple modes of toxicity; however, the persistence of some Bti toxins in the environment may increase the selection pressure for the sequential development of resistance [66]. Several mechanisms of Bti resistance have been described [67] but studies have focused on laboratory-selected strains. Few field populations have been tested, although 14 *Ae*. *aegypti* populations from Brazil in 2011 [68], 1 from Martinique [69], and 1 from Malaysia in 2008 [70] were all found to be susceptible. Both *Ae*. *aegypti* and *Ae*. *albopictus* from 6 populations in Cameroon and Gabon were tested in 2007 and found to be susceptible to Bti [71]. In contrast, there is some evidence of emerging resistance to IGRs in field populations. Recently, *Ae*. *aegypti* populations from 12 municipalities in Brazil were challenged with the IGR diflubenzuron and found to be susceptible [72] and susceptibility to pyriproxyfen and methoprene was also found in Mayotte [73], whereas a population from Martinique showed tolerance of diflubenzuron and pyriproxyfen [69], and 2 populations from the US showed some tolerance to pyriproxyfen and methoprene [74]. Furthermore, *Ae*. *aegypti* and *Ae*. *albopictus* populations from 12 states in Malaysia displayed variable levels of resistance to 5 IGRs [75].

## Conclusions

Since the geographical distribution of pyrethroid resistance was reviewed in 2010 [8], the data available has increased by more than 5-fold. The geographical spread of the data has also improved, most noticeably in West Africa, yet coverage remains extremely patchy and there is a pressing need for studies in Africa and Australasia for *Ae*. *aegypti* and in most areas for *Ae*. *albopictus*.

In order to provide a comprehensive assessment of the geographical distribution of insecticide resistance in these vectors, standard bioassay protocols need to be implemented using well-characterised susceptible reference strains. Monitoring and surveillance at a large number of locations then needs to follow. Bioassay surveillance data should also be used in conjunction with data from well-characterised markers for specific mechanisms of resistance that have been shown to be important.

Despite a growing number of studies on the mechanisms of resistance to pyrethroids and temephos, in particular, many knowledge gaps remain. Presence of VGSC mutations is unknown across large areas, especially in Africa and Australasia, which may be key foci for spreading resistance. The complexity of metabolic resistance precludes a full understanding of detoxification pathways in the near future but some key candidate genes and gene families have been identified and can be prioritised for screening or assay development. Judging the relative importance of metabolic and target-site mechanisms is difficult because of the qualitatively different nature of the 2 mechanisms. VGSC mutations affect a single gene and are easily typed, whereas metabolic resistance is much more complex with considerable potential for selection of different genes of similar function in different genetic backgrounds or environments and with variation in the specificity of the causal variants involved. Development of DNA markers for key metabolic genes will facilitate screening alongside VGSC mutations in field populations whilst controlled crosses and genome editing in the laboratory should be used to segregate and study the independent effects of each mechanism.

In addition to the standardisation of methods, development of diagnostics, and integration of phenotypic and genotypic data to improve monitoring, it is important that the resulting data are shared. Research data need to reach decision-making bodies, and health agency data held by individual countries need to be shared to address a critical global issue that is not restricted by national boundaries. Regular updates to online databases needs to be a key goal coupled with appropriate training of end users. A number of databases already exist for vector data, namely VectorBase, managed by the University of Notre Dame and Imperial College London, the Malaria Atlas Project, managed by the University of Oxford, and IR Mapper, managed by Vestergaard and the Kenya Medical Research Institute [76–78]. Incorporating the current dataset into existing repositories will make it easier to find and help ensure longer-term access to the data. We have started this process here by releasing 6,562 bioassay records, each linked to a defined time and place of mosquito collection and accompanied by all available information on how the bioassay was conducted.

**Key learning points**

- There is abundant evidence for resistance to the 2 insecticide classes most widely used to control *Ae*. *aegypti* and *Ae*. *albopictus*, pyrethroids and organophosphates, from the Americas and Asia, and some evidence for arising pyrethroid resistance in the parts of West Africa that have now been sampled.
- Evidence for the frequency and level of insecticide resistance at different times and places comes from a wide array of study methodologies and there is a critical need for internationally agreed standards coupled with surveillance in order to understand and map trends in resistance. A sensible starting point is the WHO’s test procedures for insecticide-resistance monitoring in malaria vector mosquitoes, which would need to be expanded to incorporate the range of insecticides used in the control of *Aedes* vectors.
- Insecticide resistance in *Aedes* mosquitoes is mainly due to target-site mutations and increased detoxification; however, further work is needed to identify specific mutations and genes involved in metabolic resistance and to develop robust, phenotype-calibrated diagnostics.
- Continued monitoring for AChE mutations is needed, monitoring of VGSC mutations should include but not be limited to 989P, 1016G, and 1534C in Asia and 1016I and 1534C mutations in Africa, and the frequently overexpressed metabolic genes proven to play a role (*CYP9J10*, *CYP6BB2*, *CYP9J26*, *CYP6J28*, and *CCAE3A*) should be included in the first suite of detoxification candidate genes targeted for the development of diagnostic tools.

**Top 5 papers**

1. Hirata K, Komagata O, Itokawa K, Yamamoto A, Tomita T, et al. A single crossing-over event in voltage-sensitive Na+ channel genes may cause critical failure of dengue mosquito control by insecticides. PLoS Negl Trop Dis. 2014;8: e3085.
2. Faucon F, Dusfour I, Gaude T, Navratil V, Boyer F, et al. Identifying genomic changes associated with insecticide resistance in the dengue mosquito *Aedes aegypti* by deep targeted sequencing. Genome Research. 2015;25: 1347–1359.
3. Kasai S, Komagata O, Itokawa K, Shono T, Ng LC, et al. Mechanisms of pyrethroid resistance in the dengue mosquito vector, *Aedes aegypti*: target site insensitivity, penetration, and metabolism. PLoS Negl Trop Dis. 2014;8: e2948.
4. Grigoraki L, Lagnel J, Kioulos I, Kampouraki A, Morou E, et al. Transcriptome profiling and genetic study reveal amplified carboxylesterase genes implicated in temephos resistance, in the Asian tiger mosquito *Aedes albopictus*. PLoS Negl Trop Dis. 2015;9: e0003771.
5. Ranson H, Burhani J, Lumjuan N, Black IV WC. Insecticide resistance in dengue vectors. TropIKAnet. 2010;1: 1

## Supporting information

S1 File Additional details on the bioassay data processing and mapping. (DOCX)

S2 File Dataset of bioassay records. (XLSX)

S3 File Supplementary maps showing insecticide resistance. (DOCX)

S4 File Supplementary information on markers for the mechanisms of resistance. (DOCX)

## References

[1] Global Burden of Disease Study 2013 Collaborators. Global, regional, and national incidence, prevalence, and years lived with disability for 301 acute and chronic diseases and injuries in 188 countries, 1990–2013: a systematic analysis for the Global Burden of Disease Study 2013. Lancet. 2015;386: 743–800. doi: 10.1016/S0140-6736(15)60692-4 26063472 10.1016/S0140-6736(15)60692-4 PMC4561509

[2] Naghavi M, Wang H, Lozano R, Davis A, Liang X, Zhou M, et al Global, regional, and national age-sex specific all-cause and cause-specific mortality for 240 causes of death, 1990–2013: a systematic analysis for the Global Burden of Disease Study 2013. Lancet. 2015;385: 117–71. doi: 10.1016/S0140-6736(14)61682-2 25530442 10.1016/S0140-6736(14)61682-2 PMC4340604

[3] Global Burden of Disease 2015 Disease and Injury Incidence Prevalence Collaborators. Global, regional, and national incidence, prevalence, and years lived with disability for 310 diseases and injuries, 1990–2015: a systematic analysis for the Global Burden of Disease Study 2015. Lancet. 2016;388: 1545–602. doi: 10.1016/S0140-6736(16)31678-6 27733282 10.1016/S0140-6736(16)31678-6 PMC5055577

[4] Yen C, Hyde TB, Costa AJ, Fernandez K, Tam JS, Hugonnet S, et al The development of global vaccine stockpiles. Lancet Infectious Diseases. 2015;15: 340–7. doi: 10.1016/S1473-3099(14)70999-5 25661473 10.1016/S1473-3099(14)70999-5 PMC4712379

[5] Esu E, Lenhart A, Smith L, Horstick O. Effectiveness of peridomestic space spraying with insecticide on dengue transmission; systematic review. Tropical Medicine & International Health. 2010;15: 619–31. 20214764 10.1111/j.1365-3156.2010.02489.x

[6] George L, Lenhart A, Toledo J, Lazaro A, Han WW, Velayudhan R, et al Community-effectiveness of temephos for dengue vector control: a systematic literature review. PLoS Negl Trop Dis. 2015;9: e0004006 doi: 10.1371/journal.pntd.0004006 26371470 10.1371/journal.pntd.0004006 PMC4570708

[7] Horstick O, Runge-Ranzinger S, Nathan MB, Kroeger A. Dengue vector-control services: how do they work? A systematic literature review and country case studies. Transactions of the Royal Society of Tropical Medicine and Hygiene. 2010;104: 379–86. doi: 10.1016/j.trstmh.2009.07.027 20400169 10.1016/j.trstmh.2009.07.027

[8] Ranson H, Burhani J, Lumjuan N, Black WC IV. Insecticide resistance in dengue vectors. TropIKAnet. 2010;1: 1.

[9] Corbel V, Achee NL, Chandre F, Coulibaly MB, Dusfour I, Fonseca D, et al Tracking insecticide resistance in mosquito vectors of arboviruses: the Worldwide Insecticide resistance Network (WIN). PLoS Negl Trop Dis. 2016;10: e0005054 doi: 10.1371/journal.pntd.0005054 27906961 10.1371/journal.pntd.0005054 PMC5131894

[10] Coleman M, Hemingway J, Gleave K, Wiebe A, Gething PW, Moyes CL. Developing global maps of insecticide resistance risk to improve vector control. Malaria Journal. 2017;16: 86 doi: 10.1186/s12936-017-1733-z 28222727 10.1186/s12936-017-1733-z PMC5320685

[11] Wiebe A, Longbottom J, Gleave K, Shearer FM, Sinka ME, Massey NC, et al Geographical distributions of African malaria vector sibling species and evidence for insecticide resistance. Malaria Journal. 2017;16: 85 doi: 10.1186/s12936-017-1734-y 28219387 10.1186/s12936-017-1734-y PMC5319841

[12] David J-P, Ismail HM, Chandor-Proust A, Paine MJI. Role of cytochrome P450s in insecticide resistance: impact on the control of mosquito-borne diseases and use of insecticides on Earth. Philosophical Transactions of the Royal Society B-Biological Sciences. 2013;368: 20120429. 10.1098/rstb.2012.0429 PMC3538419 23297352

[13] Hemingway J, Hawkes NJ, McCarroll L, Ranson H. The molecular basis of insecticide resistance in mosquitoes. Insect Biochemistry and Molecular Biology. 2004;34: 653–65. doi: 10.1016/j.ibmb.2004.03.018 15242706 10.1016/j.ibmb.2004.03.018

[14] Balabanidou V, Kampouraki A, MacLean M, Blomquist GJ, Tittiger C, Juarez MP, et al Cytochrome P450 associated with insecticide resistance catalyzes cuticular hydrocarbon production in *Anopheles gambiae*. Proceedings of the National Academy of Sciences of the United States of America. 2016;113: 9268–73. doi: 10.1073/pnas.1608295113 27439866 10.1073/pnas.1608295113 PMC4995928

[15] Wood OR, Hanrahan S, Coetzee M, Koekemoer LL, Brooke BD. Cuticle thickening associated with pyrethroid resistance in the major malaria vector *Anopheles funestus*. Parasites & Vectors. 2010;3. 20684757 10.1186/1756-3305-3-67 PMC2924294

[16] Davies TGE, Field LM, Usherwood PNR, Williamson MS. A comparative study of voltage-gated sodium channels in the Insecta: implications for pyrethroid resistance in Anopheline and other Neopteran species. Insect Molecular Biology. 2007;16: 361–75. doi: 10.1111/j.1365-2583.2007.00733.x 17433068 10.1111/j.1365-2583.2007.00733.x

[17] Weill M, Malcolm C, Chandre F, Mogensen K, Berthomieu A, Marquine M, et al The unique mutation in *ace-1* giving high insecticide resistance is easily detectable in mosquito vectors. Insect Molecular Biology. 2004;13: 1–7. 14728661 10.1111/j.1365-2583.2004.00452.x

[18] Grigoraki L, Lagnel J, Kioulos I, Kampouraki A, Morou E, Labbe P, et al Transcriptome profiling and genetic study reveal amplified carboxylesterase genes implicated in temephos resistance, in the Asian tiger mosquito *Aedes albopictus*. PLoS Negl Trop Dis. 2015;9: e0003771 doi: 10.1371/journal.pntd.0003771 26000638 10.1371/journal.pntd.0003771 PMC4441504

[19] Grisales N, Poupardin R, Gomez S, Fonseca-Gonzalez I, Ranson H, Lenhart A. Temephos resistance in *Aedes aegypti* in Colombia compromises dengue vector control. PLoS Negl Trop Dis. 2013;7: e2438 doi: 10.1371/journal.pntd.0002438 24069492 10.1371/journal.pntd.0002438 PMC3777894

[20] Marcombe S, Poupardin R, Darriet F, Reynaud S, Bonnet J, Strode C, et al Exploring the molecular basis of insecticide resistance in the dengue vector *Aedes aegypti*: a case study in Martinique Island (French West Indies). BMC Genomics. 2009;10: 494 doi: 10.1186/1471-2164-10-494 19857255 10.1186/1471-2164-10-494 PMC2770535

[21] Djogbenou LS, Assogba B, Essandoh J, Constant EAV, Makoutode M, Akogbeto M, et al Estimation of allele-specific *Ace-1* duplication in insecticide-resistant *Anopheles* mosquitoes from West Africa. Malaria Journal. 2015;14: 507 doi: 10.1186/s12936-015-1026-3 26682913 10.1186/s12936-015-1026-3 PMC4683970

[22] Labbe P, Berthomieu A, Berticat C, Alout H, Raymond M, Lenormand T, et al Independent duplications of the acetylcholinesterase gene conferring insecticide resistance in the mosquito *Culex pipiens*. Molecular Biology and Evolution. 2007;24: 1056–67. doi: 10.1093/molbev/msm025 17283366 10.1093/molbev/msm025

[23] Bisset J, Rodriguez MM, Fernandez D. Selection of insensitive acetylcholinesterase as a resistance mechanism in *Aedes aegypti* (Diptera: Culicidae) from Santiago de Cuba. Journal of Medical Entomology. 2006;43: 1185–9. 17162951

[24] Polson KA, Brogdon WG, Rawlins SC, Chadee DD. Characterization of insecticide resistance in Trinidadian strains of *Aedes aegypti* mosquitoes. Acta Tropica. 2011;117: 31–8. doi: 10.1016/j.actatropica.2010.09.005 20858454 10.1016/j.actatropica.2010.09.005

[25] Chen CD, Nazni WA, Lee HL, Norma-Rashid Y, Lardizabal ML, Sofian-Azirun M. Temephos resistance in field *Aedes (Stegomyia) albopictus* (Skuse) from Selangor, Malaysia. Tropical Biomedicine. 2013;30: 220–30. 23959487

[26] Lee RML, Choong CTH, Goh BPL, Ng LC, Lam-Phua SG. Bioassay and biochemical studies of the status of pirimiphos-methyl and cypermethrin resistance in *Aedes (Stegomyia) aegypti* and *Aedes (Stegomyia) albopictus* (Diptera: Culicidae) in Singapore. Tropical Biomedicine. 2014;31: 670–9. 25776592

[27] Muthusamy R, Shivakumar MS. Susceptibility status of *Aedes aegypti* (L.) (Diptera: Culicidae) to temephos from three districts of Tamil Nadu, India. Journal of Vector Borne Diseases. 2015;52: 159–65. 26119549

[28] Harris AF, Rajatileka S, Ranson H. Pyrethroid Resistance in *Aedes aegypti* from Grand Cayman. American Journal of Tropical Medicine and Hygiene. 2010;83: 277–84. doi: 10.4269/ajtmh.2010.09-0623 20682868 10.4269/ajtmh.2010.09-0623 PMC2911171

[29] Kushwah RBS, Dykes CL, Kapoor N, Adak T, Singh OP. Pyrethroid-resistance and presence of two knockdown resistance (*kdr*) mutations, F1534C and a novel mutation T1520I, in Indian *Aedes aegypti*. PLoS Negl Trop Dis. 2015;9: e3332 doi: 10.1371/journal.pntd.0003332 25569164 10.1371/journal.pntd.0003332 PMC4287524

[30] Kawada H, Higa Y, Futami K, Muranami Y, Kawashima E, Osei JHN, et al Discovery of point mutations in the voltage-gated sodium channel from African *Aedes aegypti* populations: potential phylogenetic reasons for gene introgression. PLoS Negl Trop Dis. 2016;10: e0004780 doi: 10.1371/journal.pntd.0004780 27304430 10.1371/journal.pntd.0004780 PMC4909257

[31] Martins AJ, Pereira Lima JB, Peixoto AA, Valle D. Frequency of Val1016Ile mutation in the voltage-gated sodium channel gene of *Aedes aegypti* Brazilian populations. Tropical Medicine & International Health. 2009;14: 1351–5. 19735371 10.1111/j.1365-3156.2009.02378.x

[32] Ponce Garcia G, Flores AE, Fernandez-Salas I, Saavedra-Rodriguez K, Reyes-Solis G, Lozano-Fuentes S, et al Recent rapid rise of a permethrin knock down resistance allele in *Aedes aegypti* in Mexico. PLoS Negl Trop Dis. 2009;3: e531 doi: 10.1371/journal.pntd.0000531 19829709 10.1371/journal.pntd.0000531 PMC2759509

[33] Dusfour I, Zorrilla P, Guidez A, Issaly J, Girod R, Guillaumot L, et al Deltamethrin resistance mechanisms in *Aedes aegypti* populations from three French overseas territories worldwide. PLoS Negl Trop Dis. 2015;9: e0004226 doi: 10.1371/journal.pntd.0004226 26588076 10.1371/journal.pntd.0004226 PMC4654492

[34] Linss JGB, Brito LP, Garcia GA, Araki AS, Bruno RV, Lima JBP, et al Distribution and dissemination of the Val1016Ile and Phe1534Cys *Kdr* mutations in *Aedes aegypti* Brazilian natural populations. Parasites & Vectors. 2014;7: 25. 24428880 10.1186/1756-3305-7-25 PMC3912884

[35] Vera-Maloof FZ, Saavedra-Rodriguez K, Elizondo-Quiroga AE, Lozano-Fuentes S, Black WC. Coevolution of the Ile1,016 and Cys1,534 mutations in the voltage gated sodium channel gene of *Aedes aegypti* in Mexico. PLoS Negl Trop Dis. 2015;9: e0004263 doi: 10.1371/journal.pntd.0004263 26658798 10.1371/journal.pntd.0004263 PMC4684211

[36] Du Y, Nomura Y, Satar G, Hu Z, Nauen R, He SY, et al Molecular evidence for dual pyrethroid-receptor sites on a mosquito sodium channel. Proceedings of the National Academy of Sciences of the United States of America. 2013;110: 11785–90. doi: 10.1073/pnas.1305118110 23821746 10.1073/pnas.1305118110 PMC3718148

[37] Hirata K, Komagata O, Itokawa K, Yamamoto A, Tomita T, Kasai S. A single crossing-over event in voltage-sensitive Na+ channel genes may cause critical failure of dengue mosquito control by insecticides. PLoS Negl Trop Dis. 2014;8: e3085 doi: 10.1371/journal.pntd.0003085 25166902 10.1371/journal.pntd.0003085 PMC4148226

[38] Plernsub S, Saingamsook J, Yanola J, Lumjuan N, Tippawangkosol P, Walton C, et al Temporal frequency of knockdown resistance mutations, F1534C and V1016G, in Aedes aegypti in Chiang Mai city, Thailand and the impact of the mutations on the efficiency of thermal fogging spray with pyrethroids. Acta Tropica. 2016;162: 125–32. doi: 10.1016/j.actatropica.2016.06.019 27325294 10.1016/j.actatropica.2016.06.019

[39] Saavedra-Rodriguez K, Urdaneta-Marquez L, Rajatileka S, Moulton M, Flores AE, Fernandez-Salas I, et al A mutation in the voltage-gated sodium channel gene associated with pyrethroid resistance in Latin American Aedes aegypti. Insect Molecular Biology. 2007;16: 785–98. doi: 10.1111/j.1365-2583.2007.00774.x 18093007 10.1111/j.1365-2583.2007.00774.x

[40] Yanola J, Somboon P, Walton C, Nachaiwieng W, Prapanthadara LA. A novel F1552/C1552 point mutation in the Aedes aegypti voltage-gated sodium channel gene associated with permethrin resistance. Pesticide Biochemistry and Physiology. 2010;96: 127–31.

[41] Plernsub S, Saingamsook J, Yanola J, Lumjuan N, Tippawangkosol P, Sukontason K, et al Additive effect of knockdown resistance mutations, S989P, V1016G and F1534C, in a heterozygous genotype conferring pyrethroid resistance in Aedes aegypti in Thailand. Parasites & Vectors. 2016;9. 27460671 10.1186/s13071-016-1713-0 PMC4962480

[42] Chen HY, Li KL, Wang XH, Yang XY, Lin Y, Cai F, et al First identification of *kdr* allele F1534S in VGSC gene and its association with resistance to pyrethroid insecticides in *Aedes albopictus* populations from Haikou City, Hainan Island, China. Infectious Diseases of Poverty. 2016;5: 31 doi: 10.1186/s40249-016-0125-x 27133234 10.1186/s40249-016-0125-x PMC4852438

[43] Xu JB, Bonizzoni M, Zhong DB, Zhou GF, Cai SW, Li YJ, et al Multi-country survey revealed prevalent and novel F1534S mutation in voltage-gated sodium channel (VGSC) gene in *Aedes albopictus*. PLoS Negl Trop Dis. 2016;10: e0004696 doi: 10.1371/journal.pntd.0004696 27144981 10.1371/journal.pntd.0004696 PMC4856356

[44] Liu NN. Insecticide resistance in mosquitoes: impact, mechanisms, and research directions. Annual Review of Entomology. 2015;60: 537–59. doi: 10.1146/annurev-ento-010814-020828 25564745 10.1146/annurev-ento-010814-020828

[45] Li XC, Schuler MA, Berenbaum MR. Molecular mechanisms of metabolic resistance to synthetic and natural xenobiotics. Annual Review of Entomology. 2007;52: 231–53. doi: 10.1146/annurev.ento.51.110104.151104 16925478 10.1146/annurev.ento.51.110104.151104

[46] Thompson M, Shotkoski F, ffrench-Constant R. Cloning and sequencing of the cyclodiene insecticide resistance gene from the yellow fever mosquito *Aedes aegypti*—Conservation of the gene and resistance associated mutation with *Drosophila*. Febs Letters. 1993;325: 187–90. 8391473 10.1016/0014-5793(93)81070-g

[47] Low VL, Vinnie-Siow WY, Lim YAL, Tan TK, Leong CS, Chen CD, et al First molecular genotyping of A302S mutation in the gamma aminobutyric acid (GABA) receptor in *Aedes albopictus* from Malaysia. Tropical Biomedicine. 2015;32: 554–6. 26695218

[48] Tantely ML, Tortosa P, Alout H, Berticat C, Berthomieu A, Rutee A, et al Insecticide resistance in *Culex pipiens quinquefasciatus* and *Aedes albopictus* mosquitoes from La Reunion Island. Insect Biochemistry and Molecular Biology. 2010;40: 317–24. doi: 10.1016/j.ibmb.2010.02.005 20188834 10.1016/j.ibmb.2010.02.005

[49] ffrench-Constant RH, Anthony N, Aronstein K, Rocheleau T, Stilwell G. Cyclodiene insecticide resistance: from molecular to population genetics. Annual Review of Entomology. 2000;45: 449–66. doi: 10.1146/annurev.ento.45.1.449 10761585 10.1146/annurev.ento.45.1.449

[50] Taylor-Wells J, Brooke BD, Bermudez I, Jones AK. The neonicotinoid imidacloprid, and the pyrethroid deltamethrin, are antagonists of the insect Rdl GABA receptor. Journal of Neurochemistry. 2015;135: 705–13. doi: 10.1111/jnc.13290 26296809 10.1111/jnc.13290

[51] Feyereisen R. Insect CYP Genes and P450 Enzymes. In: Gilbert LI, editor. Insect Molecular Biology and Biochemistry 2012 p. 236–316.

[52] Kasai S, Komagata O, Itokawa K, Shono T, Ng LC, Kobayashi M, et al Mechanisms of pyrethroid resistance in the dengue mosquito vector, *Aedes aegypti*: target site insensitivity, penetration, and metabolism. PLoS Negl Trop Dis. 2014;8: e2948 doi: 10.1371/journal.pntd.0002948 24945250 10.1371/journal.pntd.0002948 PMC4063723

[53] Stevenson BJ, Pignatelli P, Nikou D, Paine MJI. Pinpointing P450s associated with pyrethroid metabolism in the dengue vector, *Aedes aegypti*: developing new tools to combat insecticide resistance. PLoS Negl Trop Dis. 2012;6: e1595 doi: 10.1371/journal.pntd.0001595 22479665 10.1371/journal.pntd.0001595 PMC3313934

[54] Pavlidi N, Monastirioti M, Daborn P, Livadaras I, Van Leeuwen T, Vontas J. Transgenic expression of the *Aedes aegypti CYP9J28* confers pyrethroid resistance in *Drosophila melanogaster*. Pesticide Biochemistry and Physiology. 2012;104: 132–5.

[55] Ishak IH, Riveron JM, Ibrahim SS, Stott R, Longbottom J, Irving H, et al The Cytochrome P450 gene *CYP6P12* confers pyrethroid resistance in *kdr*-free Malaysian populations of the dengue vector *Aedes albopictus*. Scientific Reports. 2016;6: 24707 doi: 10.1038/srep24707 27094778 10.1038/srep24707 PMC4837359

[56] Faucon F, Dusfour I, Gaude T, Navratil V, Boyer F, Chandre F, et al Identifying genomic changes associated with insecticide resistance in the dengue mosquito *Aedes aegypti* by deep targeted sequencing. Genome Research. 2015;25: 1347–59. doi: 10.1101/gr.189225.115 26206155 10.1101/gr.189225.115 PMC4561493

[57] Saavedra-Rodriguez K, Strode C, Flores AE, Garcia-Luna S, Reyes-Solis G, Ranson H, et al Differential transcription profiles in *Aedes aegypti* detoxification genes after temephos selection. Insect Molecular Biology. 2014;23: 199–215. doi: 10.1111/imb.12073 24299217 10.1111/imb.12073 PMC4091897

[58] Hemingway J, Ranson H. Insecticide resistance in insect vectors of human disease. Annual Review of Entomology. 2000;45: 371–91. doi: 10.1146/annurev.ento.45.1.371 10761582 10.1146/annurev.ento.45.1.371

[59] Poupardin R, Srisukontarat W, Yunta C, Ranson H. Identification of carboxylesterase genes implicated in temephos resistance in the dengue vector *Aedes Aegypti*. PLoS Negl Trop Dis. 2014;8: e2743 doi: 10.1371/journal.pntd.0002743 24651719 10.1371/journal.pntd.0002743 PMC3961196

[60] Grigoraki L, Balabanidou V, Meristoudis C, Miridakis A, Ranson H, Swevers L, et al Functional and immunohistochemical characterization of CCEae3a, a carboxylesterase associated with temephos resistance in the major arbovirus vectors *Aedes aegypti* and *Ae*. *albopictus*. Insect Biochemistry and Molecular Biology. 2016;74: 61–7. doi: 10.1016/j.ibmb.2016.05.007 27180726 10.1016/j.ibmb.2016.05.007

[61] Chandor-Proust A, Bibby J, Regent-Kloeckner M, Roux J, Guittard-Crilat E, Poupardin R, et al The central role of mosquito cytochrome P450 CYP6Zs in insecticide detoxification revealed by functional expression and structural modelling. Biochemical Journal. 2013;455: 75–85. doi: 10.1042/BJ20130577 23844938 10.1042/BJ20130577 PMC3778711

[62] Lumjuan N, Rajatileka S, Changsom D, Wicheer J, Leelapat P, Prapanthadara LA, et al The role of the *Aedes aegypti* Epsilon glutathione transferases in conferring resistance to DDT and pyrethroid insecticides. Insect Biochemistry and Molecular Biology. 2011;41: 203–9. doi: 10.1016/j.ibmb.2010.12.005 21195177 10.1016/j.ibmb.2010.12.005

[63] Ranson H, Hemingway J. Mosquito glutathione transferases. In: Sies H, Packer L, editors. Gluthione Transferases and Gamma-Glutamyl Transpeptidases 2005 p. 226–41. 10.1016/S0076-6879(05)01014-1 16399389

[64] Noort D, van Zuylen A, Fidder A, van Ommen B, Hulst AG. Protein adduct formation by glucuronide metabolites of permethrin. Chemical Research in Toxicology. 2008;21: 1396–406. doi: 10.1021/tx8000362 18549292 10.1021/tx8000362

[65] Ismail HM, O'Neill PM, Hong DW, Finn RD, Henderson CJ, Wright AT, et al Pyrethroid activity-based probes for profiling cytochrome P450 activities associated with insecticide interactions. Proceedings of the National Academy of Sciences of the United States of America. 2013;110: 19766–71. doi: 10.1073/pnas.1320185110 24248381 10.1073/pnas.1320185110 PMC3856776

[66] Paris M, Tetreau G, Laurent F, Lelu M, Despres L, David JP. Persistence of *Bacillus thuringiensis israelensis* (*Bti*) in the environment induces resistance to multiple *Bti* toxins in mosquitoes. Pest Management Science. 2011;67: 122–8. doi: 10.1002/ps.2046 21162152 10.1002/ps.2046

[67] Melo ALD, Soccol VT, Soccol CR. *Bacillus thuringiensis*: mechanism of action, resistance, and new applications: a review. Critical Reviews in Biotechnology. 2016;36: 317–26. doi: 10.3109/07388551.2014.960793 25264571 10.3109/07388551.2014.960793

[68] Araujo AP, Diniz DFA, Helvecio E, de Barros RA, de Oliveira CMF, Ayres CFJ, et al The susceptibility of *Aedes aegypti* populations displaying temephos resistance to *Bacillus thuringiensis israelensis*: a basis for management. Parasites & Vectors. 2013;6: 297. 24499507 10.1186/1756-3305-6-297 PMC3852962

[69] Marcombe S, Darriet F, Agnew P, Etienne M, Tcha MMY, Yebakima A, et al Field efficacy of new larvicide products for control of multi-resistant *Aedes aegypti* populations in Martinique (French West Indies). American Journal of Tropical Medicine and Hygiene. 2011;84: 118–26. doi: 10.4269/ajtmh.2011.10-0335 21212213 10.4269/ajtmh.2011.10-0335 PMC3005507

[70] Loke SR, Andy-Tan WA, Benjamin S, Lee HL, Sofian-Azirun M. Susceptibility of field-collected *Aedes aegypti* (L.) (Diptera: Culicidae) to *Bacillus thuringiensis israelensis* and temephos. Tropical Biomedicine. 2010;27: 493–503. 21399591

[71] Kamgang B, Marcombe S, Chandre F, Nchoutpouen E, Nwane P, Etang J, et al Insecticide susceptibility of *Aedes aegypti* and *Aedes albopictus* in Central Africa. Parasites & Vectors. 2011;4: 79. 21575154 10.1186/1756-3305-4-79 PMC3121691

[72] Bellinato DF, Viana-Medeiros PF, Araujo SC, Martins AJ, Lima JBP, Valle D. Resistance status to the insecticides temephos, deltamethrin, and diflubenzuron in Brazilian *Aedes aegypti* populations. Biomed Research International. 2016;2016: 8603263 doi: 10.1155/2016/8603263 27419140 10.1155/2016/8603263 PMC4932163

[73] Pocquet N, Darriet F, Zumbo B, Milesi P, Thiria J, Bernard V, et al Insecticide resistance in disease vectors from Mayotte: an opportunity for integrated vector management. Parasites & Vectors. 2014;7: 299. 24984704 10.1186/1756-3305-7-299 PMC4094441

[74] Marcombe S, Farajollahi A, Healy SP, Clark GG, Fonseca DM. Insecticide resistance status of United States populations of *Aedes albopictus* and mechanisms involved. PLoS ONE. 2014;9: e101992 doi: 10.1371/journal.pone.0101992 25013910 10.1371/journal.pone.0101992 PMC4094391

[75] Lau KW, Chen CD, Lee HL, Norma-Rashid Y, Sofian-Azirun M. Evaluation of insect growth regulators against field-collected *Aedes aegypti* and *Aedes albopictus* (Diptera: Culicidae) from Malaysia. Journal of Medical Entomology. 2015;52: 199–206. doi: 10.1093/jme/tju019 26336304 10.1093/jme/tju019

[76] Giraldo-Calderon GI, Emrich SJ, MacCallum RM, Maslen G, Dialynas E, Topalis P, et al VectorBase: an updated bioinformatics resource for invertebrate vectors and other organisms related with human diseases. Nucleic Acids Research. 2015;43: D707–D13. doi: 10.1093/nar/gku1117 25510499 10.1093/nar/gku1117 PMC4383932

[77] Moyes CL, Temperley WH, Henry AJ, Burgert CR, Hay SI. Providing open access data online to advance malaria research and control. Malaria Journal. 2013;12: 161 doi: 10.1186/1475-2875-12-161 23680401 10.1186/1475-2875-12-161 PMC3662599

[78] Knox TB, Juma EO, Ochomo EO, Jamet HP, Ndungo L, Chege P, et al An online tool for mapping insecticide resistance in major *Anopheles* vectors of human malaria parasites and review of resistance status for the Afrotropical region. Parasites & Vectors. 2014;7: 76. 24559061 10.1186/1756-3305-7-76 PMC3942210

