# Correction: Contemporary status of insecticide resistance in the major Aedes vectors of arboviruses infecting humans

**DOI:** 10.1371/journal.pntd.0009084

**Published:** 2021-01-19

**Authors:** Catherine L. Moyes, John Vontas, Ademir J. Martins, Lee Ching Ng, Sin Ying Koou, Isabelle Dusfour, Kamaraju Raghavendra, João Pinto, Vincent Corbel, Jean-Philippe David, David Weetman

After the publication of this article [1] the authors noticed citation errors in Table 2. The citations for item 5 listed under pyrethroids and items 2, 3, and 4 listed under temephos refer to the wrong references and these citations have been corrected in the updated Table 2 below. The citations for items 1, 3 and 4 listed under pyrethroids and item 1 listed under temephos are also incorrect and should cite references that have been omitted from the reference list. These citations have been corrected in the updated Table 2 below and the following corresponding references 79–82 should be added to the reference list:

79 Bariami V, Jones CM, Poupardin R, Vontas J, Ranson H. Gene amplification, ABC transporters and cytochrome P450s: unraveling the molecular basis of pyrethroid resistance in the dengue vector, Aedes aegypti. PLoS Negl Trop Dis. 2012;6: e1692. pmid:22720108
80 Saavedra-Rodriguez K, Suarez AF, Salas IF, Strode C, Ranson H, Hemingway J, et al. Transcription of detoxification genes after permethrin selection in the mosquito Aedes aegypti. Insect Mol Biol. 2012;21: 61–77. pmid:22032702
81 David J-P, Faucon F, Chandor-Proust A, Poupardin R, Riaz MA, Bonin A, et al. Comparative analysis of response to selection with three insecticides in the dengue mosquito Aedes aegypti using mRNA sequencing. BMC Genomics. 2014;15: 174. pmid:24593293
82 Strode C, de Melo-Santos M, Magalhaes T, Araujo A, Ayres C. Expression profile of genes during resistance reversal in a temephos selected strain of the dengue vector, Aedes aegypti. PloS One. 2012;7: e39439. pmid: 22870187

## Updated Table

**Table 2:** The main genes identified by transcriptomic studies specifically targeting expression responses to insecticide selection or exposure.

Species	Source	Selection	RR	Genes detected as overexpressed	Ref.
		**pyrethroids**			
*Aedes aegypti*	Cuba, Cayman	historical	30–1,000^A^	CYP9J9*, CYP9J10*, CYP9J26*, CYP9J27*, CYP9J28*, CYP6BB2*, 3 other P450 or related genes*, GSTE4*, ABCB4*, 2 UGT genes*	[79]
*Ae*. *aegypti*	Singapore	10 generations	1,650^B^	CYP9M4, CYP9M5, CYP9M6, CYP6Z7, CYP6Z8, **CYP6BB2**, CYP6F2, CYP6F3, 2 other P450 or related genes	[52]
*Ae*. *aegypti*	Mexico, Peru	5 generations	2.1–10.2^A^	CYP9J28*, CYP9J32*, CYP9J9*, 8 other P450 genes*, 2 CCE genes*, 2 GST genes*, aldo-keto reductase 4118*	[80]
*Ae*. *aegypti*	Bora Bora	10 generations	3.78^C^	aldo-keto reductase 4088	[81]
*Ae*. *albopictus*	Malaysia	exposure		CYP6P12, 16 other P450 genes, 2CCE genes, 5 GST genes, 1 UGT gene, 1 aldehyde oxidase gene, 11 cuticular protein genes, multiple other gene families	[55]
		**temephos**			
*Ae*. *aegypti*	Brazil	20 generations	175^D^	**CYP6N12**, **CCEAE3A**, GSTX2, **Aldehyde oxidase AO10382**	[82]
*Ae*. *aegypti*	Thailand	exposure	5.9–9.85^E^	**CCEAE3Α****,** CCEAE6Α, 1 other CCE gene, **CYP6Z8**, **CYP9M9**, CYP6AH1, **CYP4H28**	[59]
*Ae*. *aegypti*	Colombia	exposure	15 ^A,E^	**CYP6N12**, CYP6M11, **CYP6F3**, 1 UGT gene	[19]
*Ae*. *aegypti*	Mexico, Peru	5 generations	42–390^A^	**CYP4H28, CYP6F3, CYP6Z8, CYP9M9**, 9 other P450 genes (8 CYP6 or CYP9), 8 GST genes, 10 CCE genes, **AO10382**, 7 other Redox genes	[57]
*Ae*. *albopictus*	Greece	12 generations	6.4^F^	CCEAE6A**,** **CCEAE3A**, 1 other CCE gene, CYP6M11, 7 other P450 genes, GSTX2, 1 other GST gene, 1 ABC gene, 5 UGT genes	[14]

Bold text denotes genes detected across studies; underlined text denotes genes detected across species.

The susceptible strain used to calculate the RR was New Orleans (A), SMK (B), Bora Bora (C), Rockefeller (D), Phatthalung (E) or a parental unselected line (F).

* genes significant in 2 or more comparisons.

**Abbreviations:** CCE, carboxy/cholinesterases; GST, glutathione S-transferases; P450, cytochrome P450 monooxygenases; RR, resistance ratio; Ref., Reference; UGT, UDP-glycosyltransferases.

## References

[1] Moyes CL, Vontas J, Martins AJ, Ng LC, Koou SY, Dusfour I, et al (2017) Contemporary status of insecticide resistance in the major Aedes vectors of arboviruses infecting humans. PLoS Negl Trop Dis 11(7): e0005625 10.1371/journal.pntd.0005625 28727779 PMC5518996

