# Supplementary material for: Contemporary status of insecticide resistance in the major Aedes vectors of arboviruses infecting humans
Source: PLoS Negl Trop Dis. 2017 Jul 20;11(7):e0005625. doi: 10.1371/journal.pntd.0005625 (PMC5518996; doi:10.1371/journal.pntd.0005625)
Supplement: S1 File — (DOCX) [file pntd.0005625.s001.docx]

**File S1: Bioassay data processing and mapping**

**Figure A: PRISMA Flow Diagram.** Systematic review of data source identification and data extraction.

Identification

Bioassay **records** extracted after duplicates removed

(n = 6,888)

Full-text **sources** assessed for data eligibility
(n = 249)

Data **sources** screened for data content
(n = 1,088)

Potential data **sources**
(n = 1,088)

Screening

Eligibility

Papers identified through bibliographic database search 2009 to 2016
(n = 1,077)

Additional datasets identified by the WIN*
(n = 10)

Previous literature review up to 2008
(n = 1)

Data **sources** excluded
(n = 839)

Full-text **sources** excluded
(n = 140)

***Bioassay data extraction***

The data extracted covered:- field collection dates; field site details (name, country, coordinates if provided, contextual information about the site location); species; bioassay details (generation tested, life stage tested, number tested, protocol, insecticide, concentration used, exposure duration, percent mortality and/or LC50 value); and source citation.

Geographical coordinates were converted to decimal degrees. For sites where no coordinates were given, coordinates were assigned using the site name and contextual information, such as the district and distance to a major city, using online gazeteers including GeoNames, Google Maps, Mapas da America, and OpenStreetMap. The resulting dataset was combined with the data from the 2010 review. All coordinates provided by the source and generated as part of this project were checked to ensure that they matched the sampling design described, fell on land and fell in the correct country.

***Mapping contemporary global patterns in the prevalence of resistance***

The full dataset for each insecticide class was plotted on a global map using the geographical information system software QGIS. Each dataset encompassed a range of insecticides and bioassay methods so the most widely used bioassay for each insecticide class was ascertained in order to generate maps using a comparable measure of resistance. For *Ae. aegypti* the most widely used bioassays were:

- adults challenged with propoxur (0.1% for one hour in a WHO bioassay) for the carbamates;
- adults challenged with DDT (4% for one hour in a WHO bioassay or 150µg for 30 minutes in a CDC bottle bioassay) for the organochlorines;
- larvae challenged with temephos (24 hour exposure) for the organophosphates;
- adults challenged with deltamethrin (0.05% for one hour in a WHO bioassay or 6.25µg for 30 minutes in a CDC bottle bioassay) for the pyrethroids.

Bioassay results for these test combinations were extracted and each dataset was divided into standard (for the purposes of this review, ‘standard’ refers to the most widely used and not necessarily to WHO specifications) and non-standard bioassays. If the concentration and/or exposure duration was not given, it was assumed reports that cited a published protocol used the concentration and duration recommended by that protocol. Data were also classed as being from either the last ten years (2006-15) or earlier (up to 2005). For studies that did not report which year mosquitoes were collected in, we assumed the collection year was two years before publication.

For *Ae. albopictus*, the data volumes were too low to separate out datasets for the most widely used insecticides and bioassay methods.

For temephos, all LC_50_ values were converted to mg/l. The geographical distributions of LC_50_ value and the resistance ratios obtained by each individual study conducted from 2006-2015 are shown in Fig I below. The LC_50_ values for the susceptible strains used by each study were also reviewed. The values obtained for Bora Bora and Rockefeller were the most consistent across studies worldwide and Rockefeller was the most widely tested strain (Table I below). A resistance index was therefore calculated for each field population sampled by dividing the LC_50_ value obtained for the field sample by the upper quartile value for Rockefeller obtained across studies (0.005375 mg/l).

For each class of insecticide, the full dataset for each bioassay type was displayed on a single map with the aim of showing the most recent evidence for resistance, and wherever possible, displaying data generated using consistent methods. More recent years were layered on top. Records from standard bioassays were laid over records from non-standard bioassays. If more than one record from the same location, year and standard was available, the lowest mortality value (or highest resistance index value) was displayed on top because our aim was to review evidence for resistance.

**Figure B. The geographical distribution of larval *Ae. aegypti* dose response temephos bioassays, 2006-2015**. **A.** The level of resistance expressed as the concentration required to kill 50% of the sample (LC_50_, mg/l). **B.** The resistance ratios calculated by each study split into five classes; ratios less than two and each quartile of the remaining distribution of values.


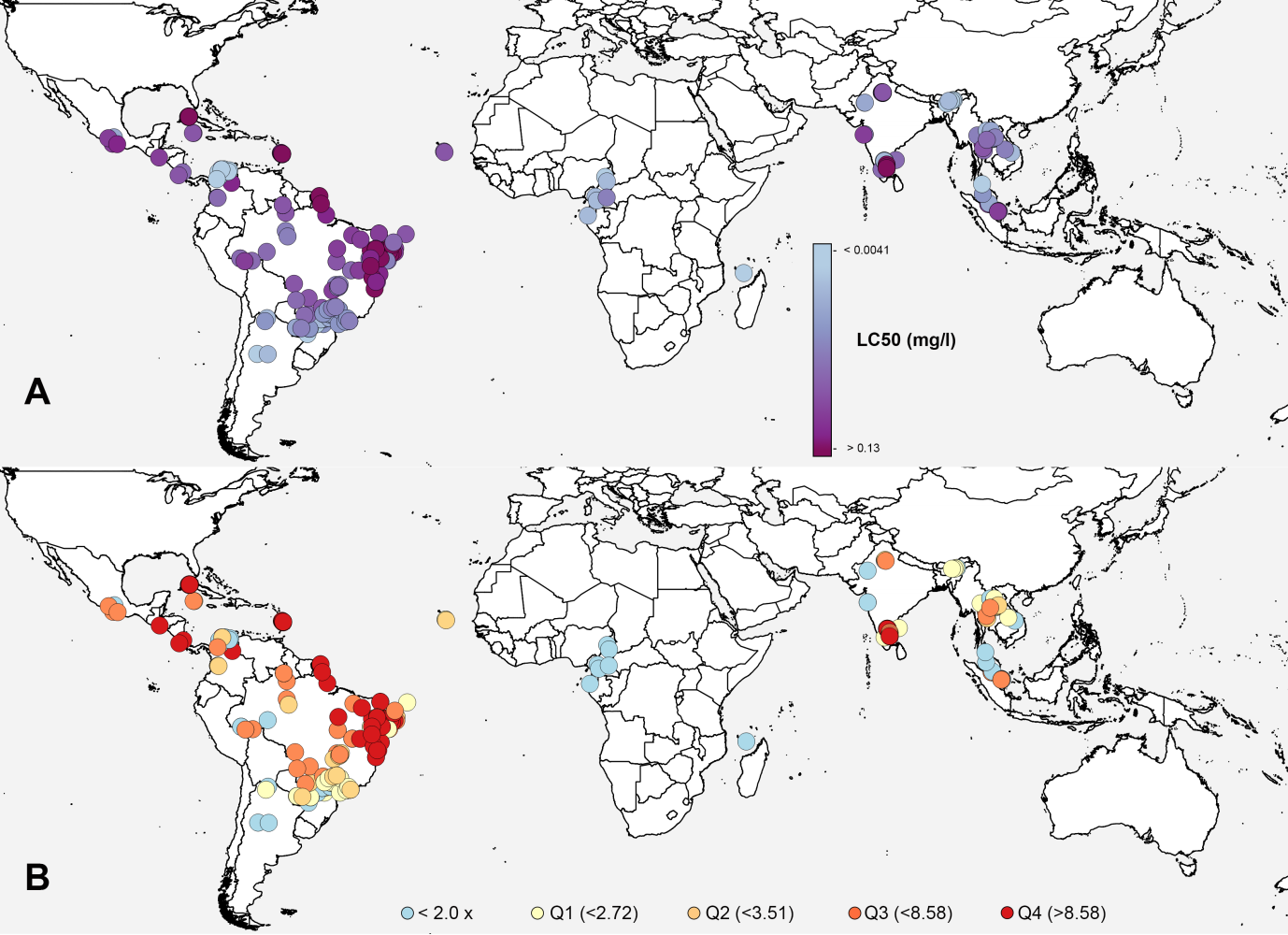


**Table A**. Concentrations required to kill 50% of each susceptible strain when challenged with temephos in studies conducted worldwide, 2006-2015.

| **Strain** | **Mean LC50, mg/l** | **N** | **Standard Deviation** | **Upper and lower 95% limits** |
| --- | --- | --- | --- | --- |
| **Bora Bora** | 0.0042 | 5 | 0.0015 | 0.0013 – 0.0071 |
| **New Orleans** | 0.0067 | 6 | 0.0040 | 0 – 0.0145 |
| **Rockefeller** | 0.0042 | 30 | 0.0027 | 0 – 0.0095 |
| **Other strains (13)** | 0.0140 | 13 | 0.0208 | 0 – 0.0548 |
