# Supplementary material for: Contemporary status of insecticide resistance in the major Aedes vectors of arboviruses infecting humans
Source: PLoS Negl Trop Dis. 2017 Jul 20;11(7):e0005625. doi: 10.1371/journal.pntd.0005625 (PMC5518996; doi:10.1371/journal.pntd.0005625)

**File S3: Supplementary Maps**

**Figure A. Distribution of contemporary and historical *Ae. aegypti* pyrethroid resistance data**. Locations of field collections used in both susceptibility assays and dose response assays are shown.

**
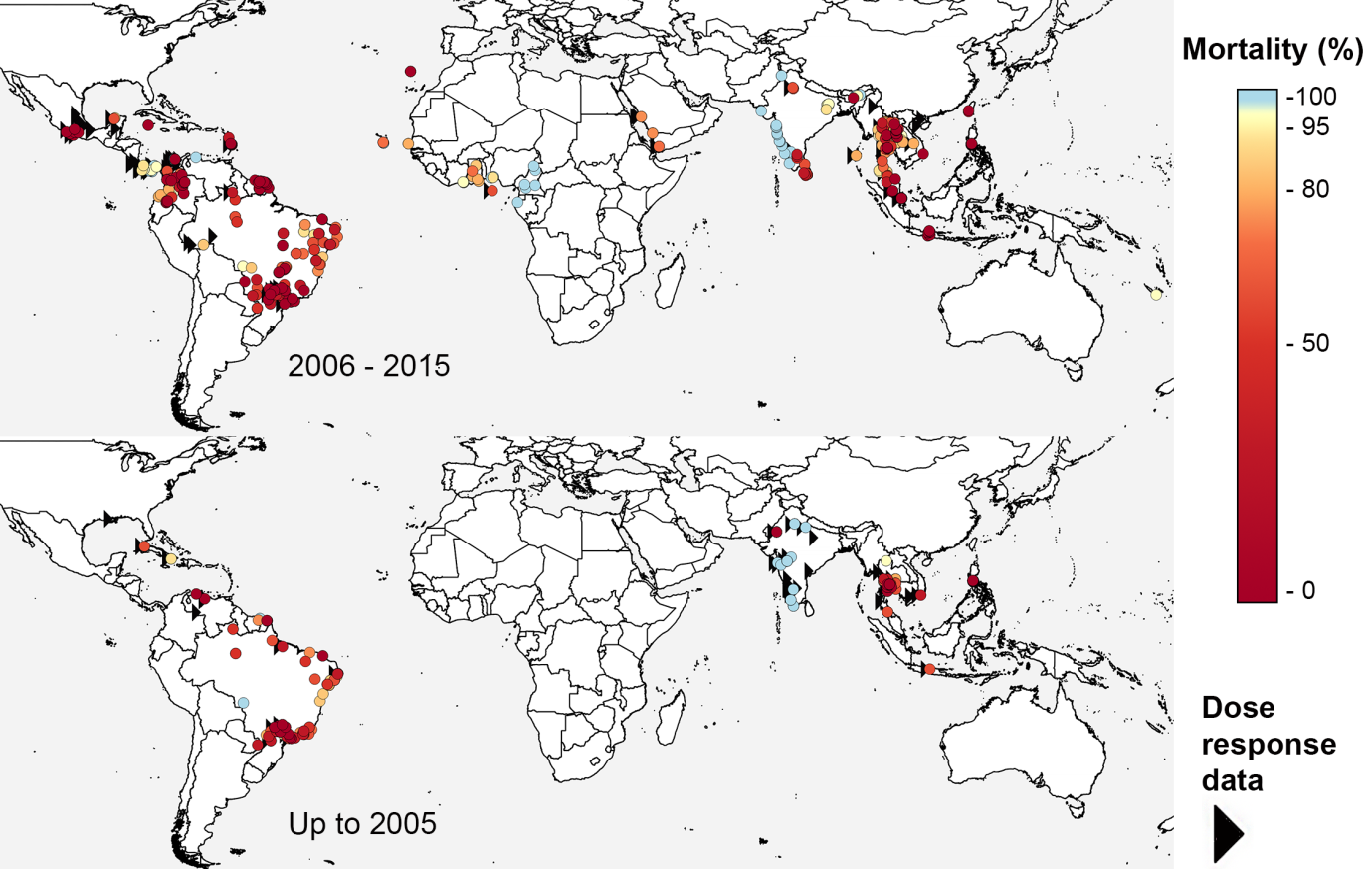
**

**Figure B**. **Frequency of organochlorine and carbamate resistance in *Aedes aegypti*, 2006 to 2015.** Distributions are shown for *Ae. aegypti* adult susceptibility bioassays using: organochlorines; the most commonly tested organochlorine, DDT (adult bioassays using 4% for one hour or CDC bottle bioassays using 150µg for 30 minutes are denoted as circles, and other adult bioassays are denoted as triangles); carbamates; and the most commonly tested carbamate, propoxur (adult bioassays using 0.05% insecticide for one hour are denoted as circles and other adult bioassays are denoted as triangles).


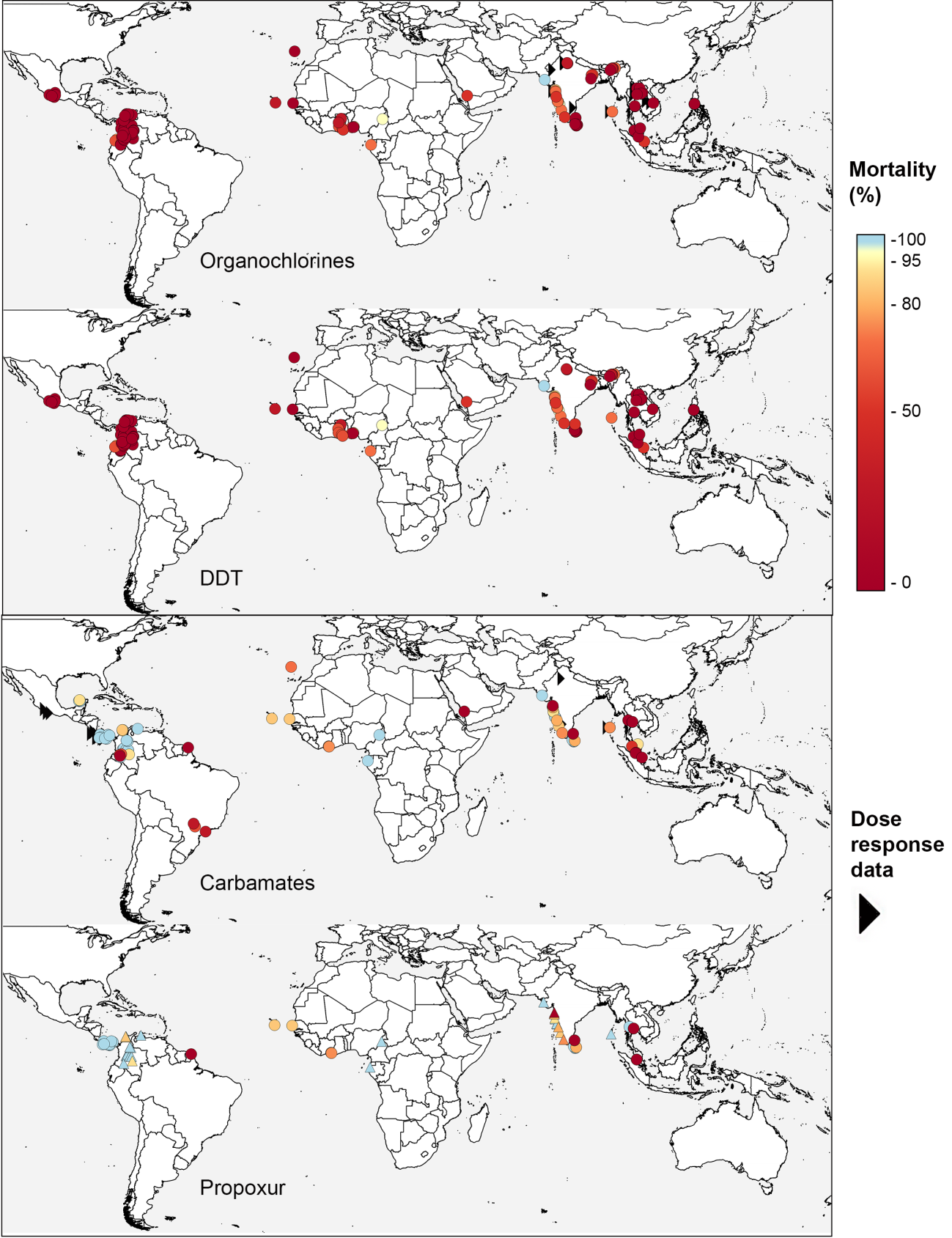

Supplement: S3 File — (DOCX) [file pntd.0005625.s003.docx]
