# Supplementary material for: Contemporary status of insecticide resistance in the major Aedes vectors of arboviruses infecting humans
Source: PLoS Negl Trop Dis. 2017 Jul 20;11(7):e0005625. doi: 10.1371/journal.pntd.0005625 (PMC5518996; doi:10.1371/journal.pntd.0005625)
Supplement: S4 File — (DOCX) [file pntd.0005625.s004.docx]

**Figure A.** **Number of resistant strains showing significant overexpression of P450 genes in *Aedes aegypti.*** Expression is shown compared to susceptible strains, based on modified and updated data from Smith *et al*. 2016 [1].


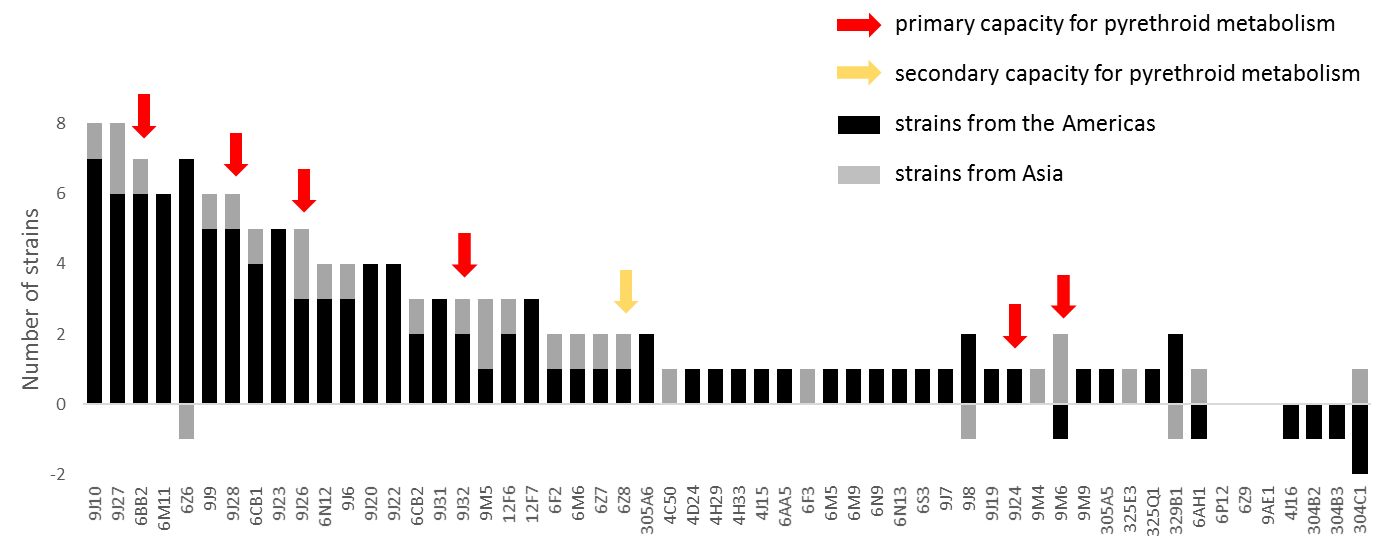


1. Smith LB, Kasai S, Scott JG (2016) Pyrethroid resistance in *Aedes aegypti* and *Aedes albopictus*: Important mosquito vectors of human diseases. Pesticide Biochemistry and Physiology 133: 1-12.

**Table A. Geographical distribution of known VGSC mutations and haplotypes.** Codons (para numbering) give wild type amino acid. See [1] for further details of mutation positions within the VGSC structure.

| IIS4-5 | IIS5-6 | IIS5-6 | IIS6 | IIIS6 | IIIS6 | IIIS6 | IVS5 | Mutation(s) | Americas | Africa | Europe | Middle East | Asia |
| --- | --- | --- | --- | --- | --- | --- | --- | --- | --- | --- | --- | --- | --- |
| *Aedes aegypti* | | | | | | | | | | | | | |
|  |  |  | V1016 |  |  |  |  | G | . | . | . |  | Thailand, Indonesia, Vietnam, Malaysia, Taiwan [2-7] |
|  |  | S989 |  |  |  |  |  | P | . | . | . |  | Indonesia [8] |
|  |  | S989 | V1016 |  |  |  |  | G+P | . | . | . | Saudi Arabia [38] | Thailand, Singapore, Myanmar, China, Indonesia [7,9-14] |
|  |  | S989 |  |  |  | F1534 |  | P+C | . | . | . |  | Indonesia [8] |
|  |  | S989 | V1016 |  |  | F1534 |  | G+P+C | . | . | . |  | Myanmar, Indonesia, Singapore [8,12,39] |
|  |  |  | V1016 |  |  | F1534 |  | G+C | . | . | . |  | Singapore, Indonesia [8,15] |
|  |  |  |  |  |  | F1534 |  | C | Venezuela, Mexico, French Guiana, Guadeloupe [16,28,29] | Ghana [17] | . | Saudi Arabia [38] | Vietnam, Thailand, Singapore, Myanmar, Indonesia, Malaysia, India [4,6,7,12,13,15,18,19] |
|  |  |  | V1016 |  |  |  |  | I | Multiple Latin American countries, Columbia, Mexico, Grand Cayman, Brazil, Venezuela, French Guiana, Guadeloupe [16,20-29] | Ghana [17] | . |  | . |
|  |  |  | V1016 |  |  | F1534 |  | I+C | Grand Cayman, Brazil, Venezuela, Mexico, French Guiana, Guadeloupe [16,23,26,28,29] | . | . |  | . |
|  |  |  | I1011 |  |  |  |  | M | Brazil, French Guiana, Guadeloupe [2,16,30,31] | . | . |  | . |
|  |  |  |  | T1520 |  | F1534 |  | I+C | . | . | . |  | India [32] |
|  |  |  | I1011 |  |  |  |  | V | Multiple Latin American countries [20] | . | . |  | Thailand [33] |
| G923 |  |  | I1011 |  |  |  |  | V+M | French Guiana, Brazil, Martinique [2] | . | . |  | . |
|  | L982 |  |  |  |  |  |  | W | . | . | . |  | Vietnam [2] |
|  |  |  | V1016 |  |  |  | D1763 | G+Y | . | . | . |  | Taiwan [3,5] |
| *Aedes albopictus* | | | | | | | | | | | | | |
|  |  |  |  |  |  | F1534 |  | C | . | . | Greece [34] | | China, Singapore [35,36] |
|  |  |  |  |  |  | F1534 |  | L | USA [37] | . | Italy [34] |  | China [34,36] |
|  |  |  |  |  |  | F1534 |  | S | USA [34] | . | . |  | China [34,36] |
|  |  |  |  |  | I1532 |  |  | T | . | . | Italy [34] |  | . |
|  |  |  |  |  |  |  |  |  |  |  |  |  |  |

1. Du Y, Nomura Y, Zhorov BS, Dong K. Sodium Channel Mutations and Pyrethroid Resistance in *Aedes* *aegypti*. Insects. 2016; 7(4): 60. doi:10.3390/insects7040060

2. Brengues C, Hawkes NJ, Chandre F, McCarroll L, Duchon S, Guillet P, et al. Pyrethroid and DDT cross-resistance in *Aedes* *aegypti* is correlated with novel mutatuions in the voltage-gated sodium channel gene. Med Vet Entomol. 2003;17: 87–94. doi:10.1046/j.1365-2915.2003.00412.x

3. Chang C, Shen WK, Wang TT, Lin YH, Hsu EL, Dai SM. A novel amino acid substitution in a voltage-gated sodium channel is associated with knockdown resistance to permethrin in *Aedes* *aegypti*. Insect Biochem Mol Biol. Elsevier Ltd; 2009;39: 272–278. doi:10.1016/j.ibmb.2009.01.001

4. Kawada H, Higa Y, Komagata O, Kasai S, Tomita T, Nguyen TY, et al. Widespread distribution of a newly found point mutation in voltage-gated sodium channel in pyrethroid-resistant *Aedes* *aegypti* populations in Vietnam. PLoS Negl Trop Dis. 2009;3: e527. doi:10.1371/journal.pntd.0000527

5. Lin Y-H, Tsen W-L, Tien N-Y, Luo Y-P. Biochemical and molecular analyses to determine pyrethroid resistance in *Aedes* *aegypti*. Pestic Biochem Physiol. Elsevier Inc.; 2013;107: 266–276. doi:10.1016/j.pestbp.2013.08.004

6. Ishak IH, Jaal Z, Ranson H, Wondji CS. Contrasting patterns of insecticide resistance and knockdown resistance (kdr) in the dengue vectors *Aedes* *aegypti* and *Aedes* *albopictus* from Malaysia. Parasit Vectors. 2015;8: 181. doi:10.1186/s13071-015-0797-2

7. Wuliandari JR, Lee SF, White VL, Tantowijoyo W, Hoffmann AA, Endersby-Harshman NM. Association between three mutations, F1565C, V1023G and S996P, in the voltage-sensitive sodium channel gene and knockdown resistance in *Aedes* *aegypti* from yogyakarta, Indonesia. Insects. 2015;6: 658–685. doi:10.3390/insects6030658

8. Sayono S, Hidayati APN, Fahri S, Sumanto D, Dharmana E, Hadisaputro S, et al. Distribution of voltage-gated sodium channel (NAV) alleles among the *Aedes* *aegypti* populations in central Java province and its aociation with resistance to pyrethroid insecticides. PLoS One. 2016;11: e0150577. doi:10.1371/journal.pone.0150577

9. Srisawat R, Komalamisra N, Eshita Y, Zheng MQ, Ono K, Itoh TQ, et al. Point mutations in domain II of the voltage-gated sodium channel gene in deltamethrin-resistant *Aedes* *aegypti* (Diptera: Culicidae). Appl Entomol Zool. 2010;45: 275–282. doi:10.1303/aez.2010.275

10. Srisawat R, Komalamisra N, Apiwathnasorn C, Paeporn P, Roytrakul S, Rongsriyam Y, et al. Field-collected permethrin-resistant *Aedes* *aegypti* from central Thailand contain point mutations in the domain IIS6 of the sodium channel gene (KDR). Southeast Asian J Trop Med Public Health. 2012;43: 1380–1386. Available: http://www.ncbi.nlm.nih.gov/pubmed/23413701

11. Kasai S, Komagata O, Itokawa K, Shono T, Ng LC, Kobayashi M, et al. Mechanisms of Pyrethroid Resistance in the Dengue Mosquito Vector, *Aedes* *aegypti*: Target Site Insensitivity, Penetration, and Metabolism. PLoS Negl Trop Dis. 2014;8: e2948. doi:10.1371/journal.pntd.0002948

12. Kawada H, Oo SZM, Thaung S, Kawashima E, Maung YNM, Thu HM, et al. Co-occurrence of Point Mutations in the Voltage-Gated Sodium Channel of Pyrethroid-Resistant *Aedes* *aegypti* Populations in Myanmar. PLoS Negl Trop Dis. 2014;8: e3032. doi:10.1371/journal.pntd.0003032

13. Li C-X, Kaufman PE, Xue R-D, Zhao M-H, Wang G, Yan T, et al. Relationship between insecticide resistance and kdr mutations in the dengue vector *Aedes* *aegypti* in Southern China. Parasit Vectors. Parasites & Vectors; 2015;8: 325. doi:10.1186/s13071-015-0933-z

14. Plernsub S, Saingamsook J, Yanola J, Lumjuan N, Tippawangkosol P, Walton C, et al. Temporal frequency of knockdown resistance mutations, F1534C and V1016G, in *Aedes* *aegypti* in Chiang Mai city, Thailand and the impact of the mutations on the efficiency of thermal fogging spray with pyrethroids. Acta Trop. Elsevier B.V.; 2016;162: 125–132. doi:10.1016/j.actatropica.2016.06.019

15. Pang SC, Chiang LP, Tan CH, Vythilingam I, Lam-Phua SG, Ng LC. Low efficacy of delthamethrin-treated net against Singapore *Aedes* *aegypti* is associated with kdr-type resistance. Trop Biomed. 2015;32: 140–150. Available: http://www.ncbi.nlm.nih.gov/pubmed/25801264

16. Dusfour I, Zorrilla P, Guidez A, Issaly J, Girod R, Guillaumot L, et al. Deltamethrin Resistance Mechanisms in *Aedes* *aegypti* Populations from Three French Overseas Territories Worldwide. PLoS Negl Trop Dis. 2015;9: e0004226. doi:10.1371/journal.pntd.0004226

17. Kawada H, Higa Y, Futami K, Muranami Y, Kawashima E, Osei JHN, et al. Discovery of Point Mutations in the Voltage-Gated Sodium Channel from African *Aedes* *aegypti* Populations: Potential Phylogenetic Reasons for Gene Introgression. PLoS Negl Trop Dis. 2016;10: e0004780. doi:10.1371/journal.pntd.0004780

18. Yanola J, Somboon P, Walton C, Nachaiwieng W, Prapanthadara L aied. A novel F1552/C1552 point mutation in the *Aedes* *aegypti* voltage-gated sodium channel gene associated with permethrin resistance. Pestic Biochem Physiol. Elsevier Inc.; 2010;96: 127–131. doi:10.1016/j.pestbp.2009.10.005

19. Muthusamy R, Shivakumar MS. Involvement of metabolic resistance and F1534C kdr mutation in the pyrethroid resistance mechanisms of *Aedes* *aegypti* in India. Acta Trop. Elsevier B.V.; 2015;148: 137–141. doi:10.1016/j.actatropica.2015.04.026

20. Saavedra-Rodriguez K, Urdaneta-Marquez L, Rajatileka S, Moulton M, Flores a E, Fernandez-Salas I, et al. A mutation in the voltage-gated sodium channel gene associated with pyrethroid resistance in Latin American *Aedes* *aegypti*. Insect Mol Biol. 2007;16: 785–798. doi:10.1111/j.1365-2583.2007.00774.x

21. García GP, Flores AE, Fernández-Salas I, Saavedra-Rodríguez K, Reyes-Solis G, Lozano-Fuentes S, et al. Recent rapid rise of a permethrin knock down resistance allele in *Aedes* *aegypti* in M??xico. PLoS Negl Trop Dis. 2009;3: e531. doi:10.1371/journal.pntd.0000531

22. Martins AJ, Lima JBP, Peixoto AA, Valle D. Frequency of Val1016Ile mutation in the voltage-gated sodium channel gene of *Aedes* *aegypti* Brazilian populations. Trop Med Int Heal. 2009;14: 1351–1355. doi:10.1111/j.1365-3156.2009.02378.x

23. Harris AF, Rajatileka S, Ranson H. Pyrethroid resistance in *Aedes* *aegypti* from Grand Cayman. Am J Trop Med Hyg. 2010;83: 277–284. doi:10.4269/ajtmh.2010.09-0623

24. Aponte HA, Penilla RP, Dzul-Manzanilla F, Che-Mendoza A, López AD, Solis F, et al. The pyrethroid resistance status and mechanisms in *Aedes* *aegypti* from the Guerrero state, Mexico. Pestic Biochem Physiol. Elsevier Inc.; 2013;107: 226–234. doi:10.1016/j.pestbp.2013.07.005

25. Brito LP, Linss JGB, Lima-Camara TN, Belinato TA, Peixoto AA, Lima JBP, et al. Assessing the Effects of *Aedes* *aegypti* kdr Mutations on Pyrethroid Resistance and Its Fitness Cost. PLoS One. 2013;8: e60878. doi:10.1371/journal.pone.0060878

26. Linss JGB, Brito LP, Garcia GA, Araki AS, Bruno RV, Lima JBP, et al. Distribution and dissemination of the Val1016Ile and Phe1534Cys Kdr mutations in *Aedes* *aegypti* Brazilian natural populations. Parasit Vectors. 2014;7: 25. doi:10.1186/1756-3305-7-25

27. Aguirre-Obando OA, Bona ACD, Duque L. JE, Navarro-Silva MA, Aguirre-Obando OA, Bona ACD, et al. Insecticide resistance and genetic variability in natural populations of *Aedes* (Stegomyia) *aegypti* (Diptera: Culicidae) from Colombia. Zool. 2015;32: 14–22. doi:10.1590/S1984-46702015000100003

28. Alvarez LC, Ponce G, Saavedra-Rodriguez K, Lopez B, Flores AE. Frequency of V1016I and F1534C mutations in the voltage-gated sodium channel gene in *Aedes* *aegypti* in Venezuela. Pest Manag Sci. 2015;71: 863–869. doi:10.1002/ps.3846

29. Vera-Maloof FZ, Saavedra-Rodriguez K, Elizondo-Quiroga AE, Lozano-Fuentes S, Black IV WC. Coevolution of the Ile1,016 and Cys1,534 Mutations in the Voltage Gated Sodium Channel Gene of *Aedes* *aegypti* in Mexico. PLoS Negl Trop Dis. 2015;9: e0004263. doi:10.1371/journal.pntd.0004263

30. AJ M, Andrade R de, Linss J, Peixoto A, Valee D. Voltage Gated Sodium Channel Polymorphism and Metabolic Resistant *Aedes* *aegypti* from Brazil. Am J Trop Med Hyg. 2009;81: 108–115. Available: https://www.ncbi.nlm.nih.gov/pubmed/19556575

31. Lima EP, Paiva MHS, de Araújo AP, da Silva EVG, da Silva UM, de Oliveira LN, et al. Insecticide resistance in *Aedes* *aegypti* populations from Ceará, Brazil. Parasit Vectors. 2011;4: 5. doi:10.1186/1756-3305-4-5

32. Kushwah RBS, Dykes CL, Kapoor N, Adak T, Singh OP. Pyrethroid-Resistance and Presence of Two Knockdown Resistance (kdr) Mutations, F1534C and a Novel Mutation T1520I, in Indian *Aedes* *aegypti*. PLoS Negl Trop Dis. 2015;9: e3332. doi:10.1371/journal.pntd.0003332

33. Rajatileka S, Black IV WC, Saavedra-Rodriguez K, Trongtokit Y, Apiwathnasorn C, McCall PJ, et al. Development and application of a simple colorimetric assay reveals widespread distribution of sodium channel mutations in Thai populations of *Aedes* *aegypti*. Acta Trop. 2008;108: 54–57. doi:10.1016/j.actatropica.2008.08.004

34. Xu J, Bonizzoni M, Zhong D, Zhou G, Cai S, Li Y, et al. Multi-country Survey Revealed Prevalent and Novel F1534S Mutation in Voltage-Gated Sodium Channel (VGSC) Gene in *Aedes* *albopictus*. PLoS Negl Trop Dis. 2016;1010: e0004696. doi:10.1371/journal.pntd.0004696

35. Kasai S, Ng LC, Lam-phua SG, Tang CS. First Detection of a Putative Knockdown Resistance Gene in Major Mosquito Vector , *Aedes* *albopictus*. Jpn J Infect Dis. 2011; 64: 217–221. Available: https://www.ncbi.nlm.nih.gov/pubmed/21617306

36. Chen H, Li K, Wang X, Yang X, Lin Y, Cai F, et al. First identification of kdr allele F1534S in VGSC gene and its association with resistance to pyrethroid insecticides in *Aedes* *albopictus* populations from Haikou City, Hainan Island, China. Infect Dis Poverty. 2016;5: 31. doi:10.1186/s40249-016-0125-x

37. Marcombe S, Farajollahi A, Healy SP, Clark GG, Fonseca DM. Insecticide resistance status of United States populations of *Aedes* *albopictus* and mechanisms involved. PLoS One. 2014;9: e101992. doi:10.1371/journal.pone.0101992

38. Al Nazawi AM, Aqili J, Alzahrani M, McCall, PJ, Weetman D. Combined target site (kdr) mutations play a primary role in highly pyrethroid resistant phenotypes of *Aedes* *aegypti* from dengue endemic areas in Saudi Arabia. Unpublished ms.

39. Kou et al. unpublished data
